# The association between mobility and crime in England and Wales

**DOI:** 10.1186/s40163-026-00291-z

**Published:** 2026-07-15

**Authors:** Hulya Seyidoglu, Jose Pina-Sánchez, Nick Malleson, Graham Farrell

**Affiliations:** 1 Gendarmerie and Coastal Guard Academy, Ankara, Turkey; 2 School of Law, University of Leeds, Leeds, UK; 3 School of Geography, University of Leeds, Leeds, UK

**Keywords:** Human mobility, Crime, Routine activities theory, COVID-19 pandemic, Mobility-crime relationship

## Abstract

**Purpose:**

This study provides the first national-level longitudinal analysis directly linking mobility data to police-recorded crime across local authorities in England and Wales, while controlling for stable structural differences between places. Its purpose is to examine how changes in human mobility are associated with changes in crime, taking advantage of the disruption in mobility caused by the COVID-19 pandemic.

**Methods:**

Negative binomial regression models were applied across 328 local authorities from March 2020 to October 2022, with area fixed effects, to assess how mobility across different activity spaces relates to police-recorded crime while accounting for unobserved, time-invariant local characteristics.

**Results:**

The exploratory and modelling analyses showed that the mobility-crime relationship varied considerably by offence type and across local authorities. Increased residential mobility was consistently associated with lower levels of property, violent, and public order crimes, while mobility around retail, grocery, transit, and workplace areas was generally associated with higher crime once stable local differences were controlled. Sensitivity analyses suggested the results are robust to plausible levels of unobserved confounding.

**Conclusion:**

The impact of mobility on crime during and after the pandemic in England and Wales was not uniform but was shaped by local conditions and time-invariant characteristics. The findings underline the need for place-specific approaches in crime prevention and suggest that mobility-related interventions should be tailored to the structural and social context of each area.

**Supplementary Information:**

The online version contains supplementary material available at 10.1186/s40163-026-00291-z.

## Introduction

The relationship between human mobility and crime has attracted increasing interest in environmental criminology. Human mobility refers to individual or aggregated patterns of movement through physical space (Browning et al., 2021). These movements reflect the spatial and temporal flows in daily life. From the Rational Choice Perspective (Cornish & Clarke, 1985, 2014), such routines generate predictable patterns that offenders can exploit or avoid. Changes in routine mobility therefore alter the distribution of opportunities for crime.

Routine Activity Theory (RAT) similarly links crime patterns to daily routines (Cohen & Felson, 1979). Crime occurs when motivated offenders encounter suitable targets in the absence of capable guardianship. As people spend more time away from home and family, their risk of victimisation increases. The COVID-19 pandemic produced an unprecedented disruption to routine activities as government restrictions limited mobility. This created a rare opportunity to observe how large-scale changes in mobility relate to changes in crime opportunities (Stickle & Felson, 2020).

In England and Wales, where the study is conducted, the first national lockdown was introduced on 23 March 2020, followed by subsequent lockdowns in November 2020 and January 2021. These restrictions were gradually eased in stages throughout spring 2021. From October 2020, the Government introduced a three-tier “Local COVID Alert Level” system, assigning different rules to each upper-tier authority. Tier 3 areas prohibited household mixing indoors and closed many venues, while Tier 1 areas were subject only to the Rule of Six (no more than six people were allowed to gather in groups). Table 1 summarises the main stages of these interventions.

**Table 1:** COVID-19 pandemic law timeline in England and Wales

Study Year	Date	Measures introduced
Year 1	Mar 2020	26 Mar: First national lockdown ordering people to stay at home
	May 2020	10 May: People who cannot work remotely return to work but not on public transport
	June 2020	01 June: Schools reopen. 15 June: non-essential shops reopen. 23 June: 2 m social distancing rule
	July 2020	04 July: Restrictions are eased in England. Pubs, restaurants, hairdressers are reopened. First local lockdown (Leicester)
	Aug 2020	03 Aug: ‘Eat out to Help Out’ hospitality subsidies. 14 Aug: Restrictions are eased further. Indoor entertainment facilities are opened
	Sept 2020	14 Sept: ‘Rule of six’ indoors and outdoors. 22 September: return to WFH; 10 pm curfew for hospitality sector
	Oct 2020	14 Oct: Three-tier area-based lockdown system
	Nov 2020	05 Nov: Second national lockdown. Schools and non-essential businesses closed. Meeting outdoor with one person outside ‘support bubble’ allowed
	Dec 2020	02 Dec: Second lockdown ends; return to three-tier lockdowns. 19 Dec: Four tier area-based lockdown system: 75% of country in tier 4 (the strictest) at turn of the year. 23–27 December: up to three households can meet up
	Jan 2021	06 Jan: Third national lockdown. Schools and non-essential businesses closed except for essential shopping. Support bubbles
	Feb 2021	15 Feb: Hotel quarantine for travellers from high-risk countries
Year 2	Mar 2021	08 March: Start of four-phase ‘roadmap out of lockdown’. 08 March, Step 1: Schools reopen. 29 March, Step 1: Stay-at-home ends. Meetings of six people or two households allowed. Sports facilities reopen
	Apr 2021	12 April, Step 2: Many non-essential businesses reopen
	May 2021	17 May, Step 3: 30 people can mix outdoors; rule of six indoors; pubs, restaurants, cinemas reopen; up to 10,000 at outdoor stadiums
	June 2021	14 June: Step 4: restrictions on weddings and funerals abolished
	July 2021	19 July: most limits on social contact removed. All sectors of economy open including nightclubs
	Dec 2021	‘Plan B’ measures introduced due to omicron variant. 10 Dec: Facemasks required in indoor venues. 15 Dec: NHS covid pass required for specific settings like nightclubs
	Feb 2022	24 Feb: Remaining pandemic restrictions removed in England
Year 3	Mar 2022	28 Mar: Remaining pandemic restrictions removed in Wales. First year without pandemic-related measures
Year 4		Second year with no pandemic-related restrictions

Several studies report that recorded crime declined sharply during the first lockdown (Dixon & Farrell, 2021; Farrell et al., 2022; Kirchmaier & Villa-Llera, 2020; Stripe, 2020). Burglary fell by roughly 25%, shoplifting by over 60%, and violence by about one-third (Langton et al., 2021a). As restrictions eased, crime trends diverged along distinct paths. National-level analyses indicate that many crime types remained below expected level (Seyidoglu et al., 2024), while local authority studies highlight substantial spatial variation linked to differences in the stringency and timing of local restrictions (Pourshir Sefidi et al., 2025).

However, most UK studies focus on broad crime trends rather than examining mobility directly. The only UK study to link crime and mobility directly is Halford et al. (2020), who introduced the Mobility Elasticity of Crime (MEC) in UK police-force area. The MEC measures how strongly crime responds to a change in human mobility. It expresses the percentage change in each crime type associated with a one per cent change in mobility, so a value near -1 indicates that crime falls by roughly the same proportion as mobility rises, whereas a value near zero indicated little response. Using this measure, they found that residential mobility was strongly associated with residential burglary (MEC ≈ -1.04), and that shoplifting and other theft responded to retail mobility (MEC = 0.84 and 0.71 respectively). Assaults, by contrast, were far less responsive. While useful, this approach aggregated daily crime counts across police-force areas and did not address fixed local differences. Such differences are important. Local authorities vary in their land-use patterns and concentrations of activity nodes such as shopping centres, entertainment venues, and transport hubs. Crime Pattern Theory describes these locations as crime generators or attractors because they concentrate potential offenders and targets (Brantingham & Brantingham, 1993). A substantial body of research shows that these relatively stable spatial features shape baseline crime risk (Browning et al., 2010; Kinney et al., 2008; Stucky & Ottensmann, 2009). Ignoring this heterogeneity may obscure how mobility changes translate into crime opportunities.

This study examines how changes in human mobility during and after the COVID-19 pandemic relate to local crime patterns in England and Wales. The analysis links Google’s Community Mobility Reports to police-recorded crime across all 328 local authorities from March 2020 to October 2022. By estimating negative binomial models with local authority fixed effects, the analysis controls for unobserved, time-invariant characteristics of local authority districts (LADs).

## Literature review

Browning et al. (2021) identified three broad approaches to understanding how mobility shapes crime opportunities. Person-centred perspectives focus on individuals’ daily routines, and their movements expose them to offending or victimisation risks. Ecological network approaches examine how flows between neighbourhoods connects places and transmit crime risks across space. Place-based and neighbourhood perspectives emphasise how local environments, land uses, and social activity patterns shape the opportunities for crime within specific locations. While these approaches differ in scale and focus, they share the assumption that human mobility structures the convergence of offenders, targets, and guardians.

Person-centred approaches emphasise how daily routines influence exposure to risky environment. Hindelang et al. (1978) demonstrated that variation in routine activities shape the likelihood of encountering potential offenders. Although measuring individua mobility at scale has traditionally been difficult, new digital data sources have begun to address this challenge. Browning et al. (2017) used GPS tracking to capture individuals’ spatial routines, linking mobility patterns to crime risk. Hipp et al. (2019) used social media traces to measure ambient presence and its predictive value for violent crime in Southern California, while Lan et al. (2023) found that Twitter activity in adjacent areas predicted theft spillovers, highlighting the utility of digital traces to approximate mobility patterns.

Ecological network approaches focus on mobility flows between areas. Rather than treating neighbourhoods as isolated units, these studies examine how movements between locations transmit risks across spatial networks. Wang et al. (2018) demonstrated that commuting between similarly disadvantaged districts in Chicago intensified homicide risks. Mondani and Rostami (2022) mapped street gang mobility across Swedish municipalities using co-offending networks, revealing spatial patterns of criminal organisation. Wardle et al. (2023) applied epidemiological models to commuting data in France and Portugal, showing how mobility networks structure spatial transmission of criminal opportunity. Together, these studies show that flows between places can shape crime risk beyond simple geographical proximity.

A major advance in place-based research has been the shift from static population to measures of the ambient population. Early studies recognised that crime risk depends on the population present, rather than resident counts (Boggs, 1965; Harries, 1995). Subsequent studies formalised ambient population denominators and demonstrated that using them changes rate estimates and spatial patterns (Andresen, 2006a, 2006b, 2011). Andresen and Jenion (2010) further showed that violence rates were nearly uncorrelated with residential populations at fine spatial scales.

More recent work employed high-frequency data to refine ambient estimates. Stults and Hasbrouck (2015) demonstrated that daily commuting rates are strong predictor of crime shifts by varying degrees across U.S. cities. Mburu and Helbich (2016) found that London’s socio-economic predictors of crime reversed once commuter flows were incorporated. Malleson and Andresen (2015, 2016) used geotagged social media data and cell-tower signals to estimate hour-by-hour population in London, finding that population flows doubled correlations with theft-from-person and shifted crime hotspots to mixed retail-residential zones. Wo et al. (2024) used social media data to recreate human mobility patterns and found association between crime counts in Los Angeles. Johnson et al., (2021a, 2021b) showed that cell-tower data changed spatial crime risk profiles in Vancouver, with downtown risks deflating while corridor risks inflated, reversing expected covariate effects. Rummens et al. (2021) used over nine million phone pings in Ghent to improve recall of bicycle-theft hotspots. Natural experiments reinforced these findings. Kurland et al. (2014) analysed event-day crime near Wembley Stadium and found spikes in raw counts but lower crime per ambient head, indicating that the venue acted as a crime generator rather than attractor. Zhang et al. (2021) showed that night-time social media heat-maps outperformed census counts for predicting burglary between 03:00 and 05:00. In Guangzhou, Zhang et al. (2022) integrated street-view imagery and ambient population to predict biweekly theft with 89% accuracy, pinpointing retail places as high-risk areas. In London, Chen et al. (2025) used footfall data and found accommodation and entertainment flows heightened theft risk, while transport-related footfall had context-dependent effects shaped by lockdown policy. Song et al. (2018) found that residential population estimates crime risk better than other measures in the morning while taxi ridership and phone users are better indicators in the afternoon and evening. Together, these studies demonstrated that crime risk is better explained by dynamic measures of human presence and mobility than residential population. Yet, Okmi et al. (2023) highlighted in their systematic review, most ambient population research remains cross-sectional and descriptive, relying on spatial clustering rather than longitudinal designs capable of identifying causal relationships.

Structural features of place can shape baseline crime rates and thus must be treated as time-invariant characteristics in longitudinal analyses. Research on land use demonstrates that commercial zones, transit infrastructure, and mixed-use developments serve as crime generators, owing to the volume of unstructured footfall they attract and the inconsistent levels of surveillance they entail (Anderson et al., 2013; Zahnow, 2018). Building on this, Eck and Weisburd (2015) theorise that crime generators increase opportunities simply by assembling large crowds for legitimate purposes, while crime attractors lure motivated offenders who exploit these opportunity-rich environments. Kennedy et al. (2011) exemplify this with transit stations, which often function simultaneously as generators and attractors, and crowded spaces where the routine convergence of potential offenders and suitable targets occurs with minimal guardianship. Tillyer et al. (2021) refine this view with their “place-in-neighbourhood” model, showing that the criminogenic influence of such facilities is contingent on local context: facilities like bars or convenience stores elevate violent and property crime most acutely in socioeconomically disadvantaged or disorganised areas. These studies collectively reinforce the idea that place-based structural conditions shape crime opportunity, underscoring the importance of controlling the heterogeneity between places.

Several studies investigated impact of mobility change on crime during the COVID-19 pandemic. Situ (2024) demonstrated that Baltimore census tracts with collapsed inward flows saw sharp declines in gun violence. Valente (2024) found that Barcelona’s walkable blocks with high transient populations experienced elevated theft and robbery. Venverloo et al. (2023) combined computer vision, GPS tracking, and spatial analytics to uncover micro-scale bike theft markets in Amsterdam. Cheung and Gunby (2022) observed significant declines in property and personal crimes across New Zealand during lockdowns, attributing these drops to reduced situational opportunities outside the home. Çalışkan (2024) reported similar trends in Türkiye, linking dramatic mobility reductions using Google mobility data during lockdown to substantial decreases in theft and assault but not homicide. Nikolovska et al. (2020) found in the UK that decreased transit mobility led to reduced street crimes but increased online fraud, emphasizing different crime dynamics in physical and digital spaces. Paramasivan et al. (2024) used transport data and Google mobility data in Tamil Nadu, India to confirm substantial declines in violent crimes aligned closely with decreased mobility around transit stations, reinforcing the guardianship dimension of place-based theory. Their earlier work similarly showed pronounced drops in property crimes under lockdown, clearly connecting mobility patterns to crime opportunity structures (Paramasivan et al., 2022). Gu et al. (2025) found that lockdown measures had a varied impact on mobility among different racial groups in Cincinnati, Ohio. Furthermore, they also found statistically significant link between street crimes and the ambient population.

Taken together, this literature highlights four key developments. First, studies increasingly replace static resident populations with dynamic measures of who present in a place. Second, they move beyond head counts to examine crowd composition and timing of crowds. Third, they incorporate insights from guardianship theory to understand how design features and activity patterns shape opportunities for intervention. Fourth, the COVID-19 pandemic created a unique setting for observing how large-scale disruptions to mobility affect crime. Despite these advances, Okmi et al. (2023) noted that most ambient population studies remain cross-sectional and descriptive. Therefore, three important gaps remain. First, most studies focused on single cities or short time periods, leaving lack of national longitudinal evidence on mobility and crime. Second, studies using panel data often fail to control for the stable, structural differences between places. Third, Google’s Community Mobility Reports have rarely been linked to police-recorded crime across local authorities in England and Wales. Addressing these gaps allows mobility to be examined not only as a descriptive indicator of human presence but also as a behavioural mechanism through which disruptions to routine activities may translate into changes in crime opportunities.

Drawing on Routine Activity Theory, we expect that when people spend more time at or near home, burglary will decline (H1), because increased household presence strengthens informal guardianship. Conversely, when more people gather at transport hubs, theft from the person will rise (H2), due to a higher concentration of suitable targets and low levels of guardianship in these spaces. We also expect that mobility linked to workplaces will increase incidents of violence and sexual offences more than mobility linked to retail and leisure areas (H3), because convergence between offenders and targets is more sustained and potentially less supervised in work settings. Finally, we predict that retail-related mobility will increase shoplifting and theft from the person more sharply than robbery (H4), since the former crimes benefit from crowded, distracting environments, while robbery involves higher risks from potential witnesses and confrontation. We used police recorded crime data and Google’s Community Mobility Reports to test the hypotheses.

## Methods

### Data

This study draws on police-recorded crime data in England and Wales^1^, covering thirteen crime categories and antisocial behaviour (ASB). These categories follow the Home Office Crime Recording Rules, which combine individual notifiable offences into larger categories (Table 2). Because several categories combine multiple offences, the implications of this aggregation for interpreting the results are discussed later. To align crime data with Google’s Community Mobility Reports (GMR), crime data were aggregated from lower layer super output areas to LAD boundaries using and Office for National Statistics^2^ geography lookup table. The final dataset covers 328 LADs observed monthly from March 2020 to October 2022. England contains 317 LADs, and there are 22 unitary authorities, in Wales^3^.

**Table 2:** Crime categories

Crime type	Explanation
All crime	Total for all categories
ASB	Individual, environmental and nuisance anti-social behaviour
Bicycle theft	Taking of a pedal cycle without consent or theft of a pedal cycle
Burglary	Entering a house or a building with the intention of stealing
Criminal damage and arson	Damage to buildings and vehicles and deliberate damage by fire
Drugs	Offences related to supply, possession, and productions of drugs
Other crime	Forgery, perjury, and other miscellaneous crime
Other theft	Theft by an employee, blackmail and making off without payment
Possession of weapons	Possession of firearms or knives
Public order	Offences causing fear, alarm, or distress of public
Robbery	Using force or threat of force to steal
Shoplifting	Theft from shops or stalls
Theft from the person	Theft directly from victim without force of threat of force
Vehicle crime	Theft from or of a vehicle or interfering in a vehicle to steal the vehicle or steal items in a vehicle
Violence and sexual offences	Offenses against the person include regular assaults, bodily harm, and sexual offences

Taken from https://www.police.uk/pu/about-police.uk-crime-data/

Greater Manchester was excluded because its ten authorities did not release crime data during the study period, and then the Isle of Scilly was removed because mobility data were unavailable. Following the spatial aggregation, monthly crime counts were calculated for each LAD across the study period.

GMR data tracks daily changes in the number of people who enabled location history on their Google Account in different place categories relative to a pre-pandemic baseline. The baseline period covers the 5-week span from January 3 to February 6, 2020. The place categories are retail and recreation, grocery and pharmacy, parks, transit stations, workplaces, and residential areas (Table 3). The values represent percentage changes relative to the median value for the same day of the week during the baseline period. Since daily time is constrained to 24 h, increases in time spent in other categories naturally reduce residential time. For example, a decrease in ambient population at parks or transit stations on weekends during lockdowns may have little effect if those locations were already less visited on those days (Google, 2020).

**Table 3:** Place categories recorded in Google community mobility reports

Place category	Description
Retail & Recreation	Locations such as restaurants, cafes, shopping centres, theme parks, museums, libraries, and movie theatres
Grocery & Pharmacy	Places like grocery markets, food warehouses, farmers markets, specialty food shops, drug stores, and pharmacies
Parks	Public gardens, parks, national parks, public beaches, marinas, dog parks, plazas, and public gardens
Transit Stations	Public transport hubs such as subway, bus, and train stations
Workplaces	Places of work excluding remote work locations
Residential	Areas where people live, including their homes

Taken from: https://www.google.com/covid19/mobility/data_documentation.html?hl=en-GB, accessed on 05 March 2025

GMR reports observations at both regional and Lad levels for England and Wales. Most LAD identifiers in the mobility data follow the April 2019 ONS boundary set, but some records reflect the earlier 2018 configuration. To create a consistent panel, the Data Science Campus LAD-lookup table^4^ was used to match each Google record to its 2019 boundaries.

GMR dataset includes intentional gaps where privacy or data-quality standards are not met. Specifically, Google omits data for any geographic area smaller than roughly 3 km2 or for any day and place where fewer than 100 users contributed anonymized location information, to ensure that no individual can be re-identified. In addition, Google applies differential privacy by adding random noise to all reported mobility values. Observations are suppressed when this noise produce changes exceeding a 10% threshold (Aktay et al., 2020). Consequently, some LAD-day combinations are missing, not because mobility was unmeasurable, but because Google’s privacy filters excluded them. Missingness in the mobility data was examined descriptively across space and time. Missing observations unevenly distributed over time or space, with higher levels concentrated in specific months and particularly in parks category. This pattern likely reflects lower user density in certain activity spaces and the operation of Google’s privacy filters^5^.

Except for parks, missing observations accounts for less than 5% of total observations (Table 4). Parks exhibit higher missingness (17.7%), likely due to lower user density in such locations. Missing mobility values were imputed using multiple imputation by chained equations (Rubin, 2004; Van Buuren, 2018). Specifically, we use the *mice* package in R, predictive mean matching (Schafer, 1997), and five imputed datasets generated over 20. The imputation model included population size, daily COVID‑19 case counts, day‑of‑week and month-of-year indicators (see Johnson et al., (2021a, 2021b)) and local authority fixed effect, capturing observed temporal and spatial structures associated with missingness. Since police-recorded crime data are available only at monthly intervals at the local authority level, both crime and mobility measures were aggregated to the month. This temporal aggregation limits the ability to examine short-term sequencing between mobility and crime within a given month. As a result, the models estimate associations between monthly changes in mobility and monthly crime counts, rather than testing whether mobility changes precede crime changes at finer temporal scales.

**Table 4:** Missing daily observations in Google's community mobility reports

Place categories	Missing Obs	% of Missing Obs	N
Retail & recreation	7253	1.97	366,339
Grocery & pharmacy	7773	2.12	366,339
Parks	64,877	17.70	366,339
Transit stations	7007	1.91	366,339
Workplaces	1625	0.44	366,339
Residential	14,757	4.02	366,339

### Analytical strategy

The study estimates the association between mobility and crime at LAD level. Negative binomial models are used as they provide the best fit to right skewed over-dispersed crime rates^6^. To control for unobserved, time-invariant heterogeneity across LADs, an area fixed-effects term is included in each specification. By conditioning on each LAD’s own mean, the fixed‑effects estimator removes stable, time-invariant differences between local authorities, such as historically high-crime profiles, long-standing socio‑economic structure, or persistent land‑use composition that are not directly measured. The logic is that between-area differences that remain constant over the observation period are absorbed by the LAD-specific intercept, allowing the estimates to reflect with-in area changes over time. For every crime category *c* and each Google mobility stream *m*, we estimate a negative-binomial area-fixed-effects (AFE) model:

1 $$\begin{array}{*{20}c} {log\left( {\mu_{at}^{\left( c \right)} } \right) = \alpha_{a} + \beta_{m}^{\left( c \right)} \,X_{at}^{\left( m \right)} + logP_{at} ,\quad \quad Y_{at}^{\left( c \right)} \sim {\mathrm{NB}}\left( {\mu_{at}^{\left( c \right)} ,\,\theta^{\left( c \right)} } \right)} \\ \end{array}$$

where ${Y}_{at}^{\left(c\right)}$ is the monthly count of offence $c$ in local authority $\alpha$ at month $t$; ${X}_{at}^{\left(m\right)}$ is the corresponding percentage change in mobility $m$; ${\alpha}_{a}$ is a LAD-specific intercept implemented as dummy variable absorbing all time-invariant attributes of place; ${\beta}_{m}^{\left(c\right)}$ is the common within-area effect of that mobility stream on offence $c$; $P$ is monthly population for each LAD; and ${\theta }^{\left(c\right)}$ is the over-dispersion parameter^7^. We applied the Benjamini–Hochberg false discovery rate correction separately within each model specification to account for the multiple mobility-crime tests.

Separate models are estimated for each of the six mobility categories and fourteen crime categories. Because the six mobility indicators capture overlapping aspects of routine activity, they are strongly correlated with one another^8^. Including mobility indicators in a single specification would therefore introduce substantial multicollinearity. The coefficients should therefore be interpreted as associations between a specific mobility category and crime, rather than as net effects conditional on all other mobility types.

The value of area fixed effects (AFE) becomes clear when contrasted with a simple regression that fits one line to all observations. A pooled model imposes a single intercept and slope across all areas, mixing differences between places with changes within places. This can distort the estimated relationship, particularly when areas differ substantially in baseline crime levels, producing overestimates in some and underestimates in others, and in extreme cases it can even reverse the apparent direction of the relationship (Carilli, 2021). AFE removes time-invariant area differences by giving each area its own baseline, so the estimated association primarily reflects change within the same area over time, conditional on the assumption that relevant confounders are stable.

To facilitate comparison across crime types and mobility indicators, we standardised coefficients by following Schielzeth (2010). Each coefficient is rescaled to represent the expected change in the log crime count with a one-standard-deviation change in the mobility indicator relative to the dispersion of the outcome:

2 $$\begin{array}{*{20}c} {\beta_{{{\mathrm{std}}}} = \beta_{{{\mathrm{unstd}}}} \times \left( {\frac{{\sigma_{X} }}{{\sigma_{{\log \left( {Y + 1} \right)}} }}} \right)} \\ \end{array}$$

where ${\upsigma}_{X}$ is the standard deviation of the mobility indicator and ${\upsigma}_{\mathrm{log}\left(Y+1\right)}$ is the standard deviation of the log-transformed monthly crime count for that offence. The resulting ${\upbeta}_{\mathrm{std}}$ values are directly comparable across models. Finally, residential mobility category differs from the other mobility measures. Mobility changes in all categories except residential represent percentage shifts in the number of people visiting those locations relative to pre-pandemic baseline. However, the residential mobility reflects percentage changes in the amount of *additional* time spend at home. Here, ${x}_{i}$ is the observed time spent at home, and ${b}_{i}$ is the baseline.:

3 $$\begin{array}{*{20}c} {{\text{residential change (\% )}} = \frac{{x_{i} - b_{i} }}{{24 - b_{i} }} \times 100} \\ \end{array}$$

For example, suppose the baseline daily median time spent at home is 8 h in a LAD and increased to 24 h, during full lockdowns. This makes 100% increase in time spent at home. So, even small percentage changes (6%, for example) could mean additional 1 h spent at home.

## Mobility trends

Mobility changed sharply during the first year of the pandemic. Retail and recreation, transit stations, and workplaces experienced the largest reductions, with average decreases of 42%, 41.2%, and 39.2%, respectively. Residential mobility increased by 14.5%. Parks increased average 24% during the first year (Table 5). By May 2020, those who could not work remotely were encouraged to return to workplaces, therefore, workplace mobility slightly recovered. However, average change in workplace mobility in the first year of the pandemic is -39.2%, which is well below the baseline. The reopening of non-essential shops and hospitality increased retail and recreation mobility to around -20% below the baseline by July. In August 2020, the “Eat Out to Help Out” scheme further stimulated visits to in leisure-related categories.

**Table 5:** Mean monthly change (%) from median baseline values (Jan 3–Feb 6, 2020)

Mobility category	1st year (Mar 20–Feb 21)	2nd year (Mar 22–Feb 22)	3rd year (Mar 22–Oct 22)
Retail & recreation	− 42.0	− 12.3	− 4.14
Grocery & pharmacy	− 13.0	5.1	9.2
parks	24.0	43.2	47.9
Transit stations	− 41.2	− 26.8	− 19.2
Workplaces	− 39.2	− 27.5	− 22.6
Residential	14.5	7.8	4.1

**Fig. 1:**
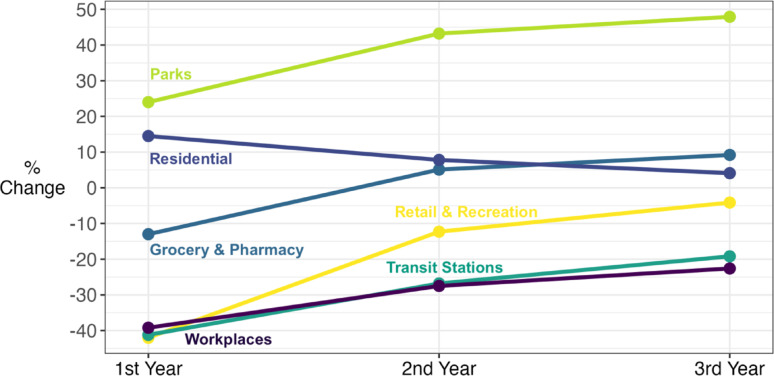
Mean monthly change from median baseline values for six mobility categories

Subsequent lockdowns produced renewed mobility reductions. During the second national lockdown in November 2020, retail and recreation mobility fell to roughly 50% below baseline and transit station mobility to around 45% below baseline. Residential mobility rose again to approximately 25% above baseline. The third lockdown in January 2021 produced similar patterns, with transit and workplace mobility falling to about 50% below baseline while residential mobility remained elevated (Fig. 1).

**Fig. 2:**
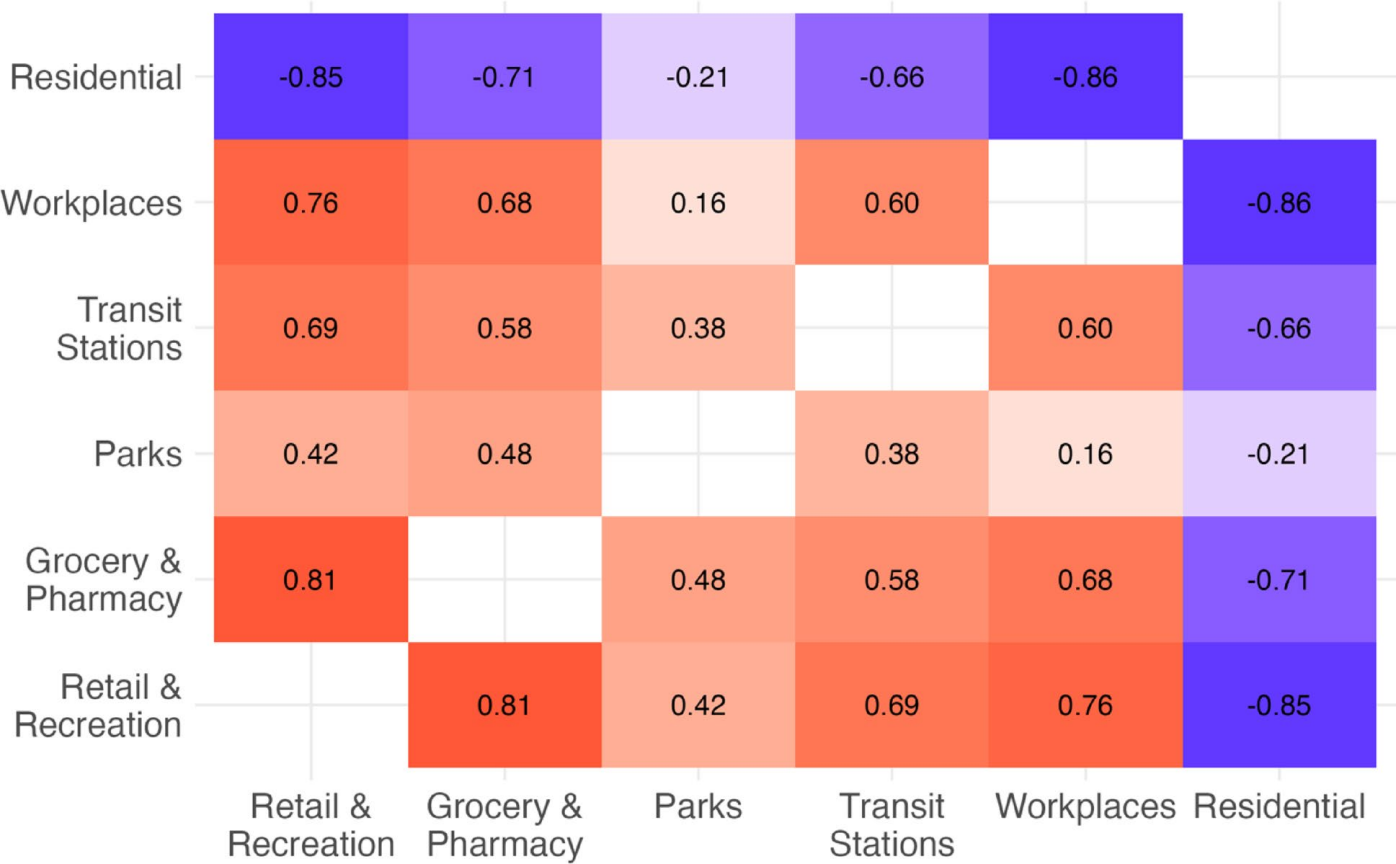
Correlation heatmap of mobility indicators

These trends also reveal deeper interdependencies between different mobility categories. A strong positive correlation between most of the mobility categories was found. For example, mobility in retail & recreation areas shows high correlations with grocery & pharmacy stores (0.80), transit stations (0.69) and workplaces (0.76), suggesting that visits to retail locations often occur alongside shopping, commuting, and work-related activities. Conversely, residential mobility exhibits strong negative correlations with retail & recreation (− 0.85), grocery & pharmacy (− 0.70), transit stations (− 0.64), and workplaces (− 0.88) (Fig. 2). This confirms the expected inverse relationships. As people spend more time at home, their visits to workplaces, transit hubs, and shopping areas decrease.

## Results

**Fig. 3:**
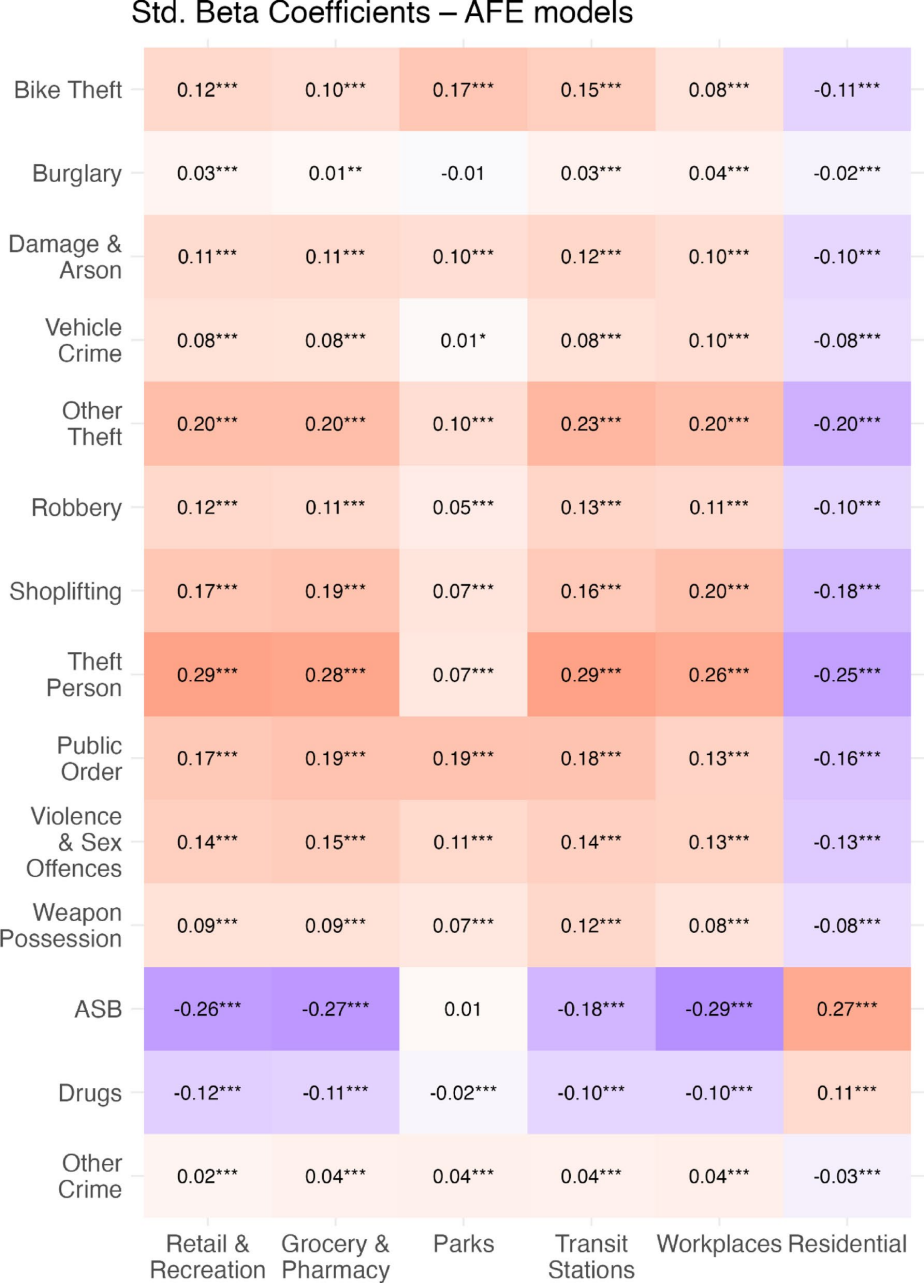
Std. beta coefficients of mobility categories from NB regression with fixed effect model * *p* < .05, ** *p* < .01, *** *p* < .001 (Statistical significance for Fig. 3 and Fig. 4: * *p* < .05, ** *p* < .01, *** *p* < .001)

**Fig. 4:**
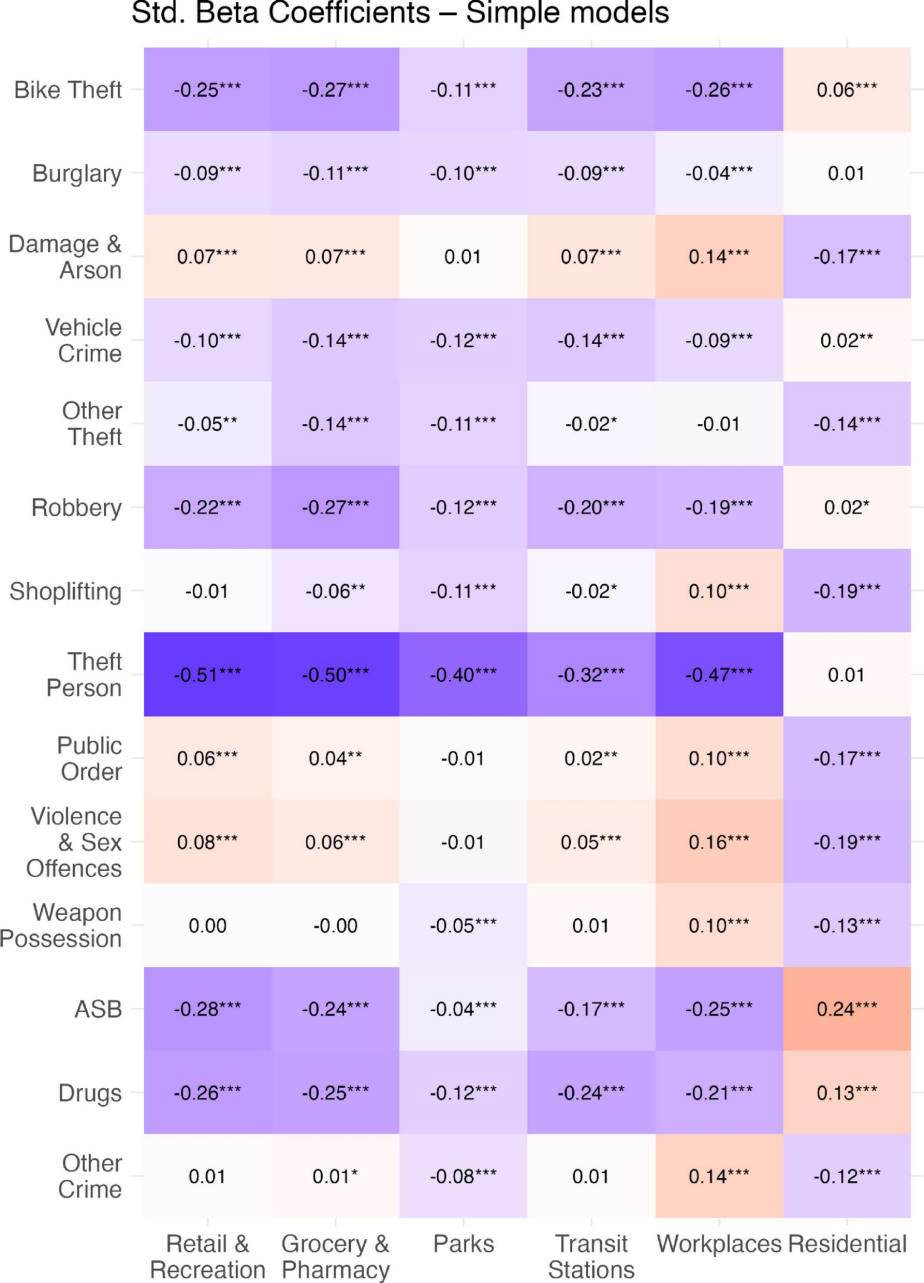
Std. beta coefficients of mobility categories from NB regression model from simple regression model without fixed effect

We estimated 84 negative binomial regression models linking six mobility indicators to thirteen police-recorded crime and one anti-social behaviour. The main specification includes local authority area fixed effect (AFE), allowing the estimates to reflect with-in area mobility changes overtime. Figure 3 presents the standardised coefficients from these models while Fig. 4 visualise std Beta coefficients from simple regression, serving as a benchmark. Across the 84 coefficients, 82 remain statistically significant after applying the Benjamini–Hochberg false discovery rate correction. The goal is not interpreting every coefficient individually but discern systematic relationship between mobility and crime.

We found no substantively meaningful association between residential mobility and burglary; the effect size is very small even were statistically significant (β_std_ = − 0.02, *p* < 0.001, Fig. 3). Thus, H1 is not supported and burglary responses to residential mobility are negligible. However, residential mobility shows negative associations with most crime categories, particularly shoplifting (β_std_ = − 0.18), theft from the person (β_std_ = − 0.25), and other theft (β_std_ = − 0.20). Consistent with H2, transit-station mobility is strongly associated with theft from the person (β_std_ = 0.29, *p* < 0.001, Fig. 3). Retail and recreation mobility shows a similarly large association (β_std_ = 0.29, *p* < 0.001). In contrast, robbery shows a smaller association with retail mobility (βstd = 0.12). The results do not support H3. Workplace mobility is positively associated with violence and sexual offences (β_std_ = 0.13, *p* < 0.001), but its magnitude is similar to other mobility indicators, including retail & recreation (β_std_ = 0.14) and grocery & pharmacy (β_std_ = 0.15), and nearly identical to transit (β_std_ = 0.14) and parks (β_std_ = 0.11).

Park mobility displays comparatively small coefficients across most offences. The largest association appear for public order (β_std_ = 0.19, *p* < 0.001) and violence and sexual offences (β_std_ = 0.11, *p* < 0.001), while the remaining estimates are close to zero (Fig. 3).

For comparison, Fig. 4 reports results from simple pooled models without fixed effects. In these models, many coefficients change direction, reflecting differences between areas rather than within-area changes. This contrast illustrates the importance of controlling for time-invariant local characteristics when estimating mobility-crime relationships. We also estimated models including month fixed effects. Introducing time fixed effects reduces the magnitude of most coefficients while leaving the direction of associations largely unchanged. This reduction reflects the fact that much of the variation in mobility occurs at the monthly level during the pandemic. Full results from the time fixed-effects models are reported in the Appendix Figs. 4A–5A.

We next assessed the robustness of selected mobility-crime associations to confounding bias using the robustness value approach proposed by Cinelli and Hazlett (2020). Because this method is designed for linear models, we replicated a subset of models using linear regression with log-transformed crime counts while retaining local authority fixed effects and the population offset. Four theoretically relevant relationships were examined: transit mobility and theft from the person, retail mobility and theft from the person, retail mobility and shoplifting, and retail mobility and robbery. The robustness values for the four combinations of mobility indicator and offence type considered range from 0.372 (for transit mobility and theft from the person) to 0.182 (for retail mobility and robbery).

This means that even for the weakest of the associations considered, a hypothetical unobserved confounder would need to explain, simultaneously, more than 18.2% of the residual variance in both the mobility indicator and the offence type to eliminate the observed effect (31.5% to render it statistically non-significant).

## Discussion

Periods of strict pandemic restrictions corresponded with lower mobility in public spaces and increased time spent at residential areas. During these periods, several crime categories declined, consistent with reduced opportunities for interaction and target availability in public spaces. As restrictions eased and mobility gradually recovered, activity in public spaces increased and crime patterns began to diverge across offence types. The results align with previous findings showing that the pandemic produced sudden and spatially heterogeneous changes in mobility (Santana et al., 2023; Shepherd et al., 2021).

Comparing results across model specifications highlights the importance of accounting for spatial heterogeneity. National-level patterns obtained from pooled models often differ from those estimated using local authority fixed effects. Pooled models combine between-area differences with within-area change, which can obscure or even reverse the relationship. In contrast, AFE models allow each LAD has its own baseline level of crime, so the estimates reflect how mobility changes withing the same area over time rather than differences between areas.

When month fixed effects are included, effect sizes decline but remain largely consistent in direction. This suggests that part of the association reflects national mobility shocks that affected all areas simultaneously, while the remaining variation reflects differences in mobility change across LADs within the same months. It is important to emphasise that the models are designed to estimate associations between mobility and crime rather than to identify a fully specified causal pathway. Pandemic-related factors, such as changes in economic disruptions, changes in policing practices, or public health intensity, may also have influenced crime trends during this period. Some of these factors may act both as confounders and as mediators of the relationship between pandemic conditions, mobility, and crime. This dual role complicates the identification strategy, as controlling for such variables may introduce post-treatment bias if they lie on the causal pathway (Cinelli et al., 2024). For this reason, we focus on the reduced-form association between mobility and crime, while acknowledging that additional time-varying factors beyond mobility may contribute to observed changes. We also consider sensitivity analysis based on the robustness value further ahead.

To assess the extent to which omitted time-varying factors could account for the observed relationships, we conducted a sensitivity analysis using the robustness value approach proposed by Cinelli and Hazlett (2020). Focusing on several theoretically central associations, the results indicate that the strongest relationships are relatively robust to unobserved confounding. For example, the association between transit mobility and theft from the person would require an unobserved factor explaining roughly 33% of the residual variance in both the predictor and the outcome to fully eliminate the estimated effect. Similar thresholds were observed for retail mobility with theft from the person (RV = 0.372) and shoplifting (RV = 0.356). By contrast, the retail mobility-robbery relationship is more sensitive (RV = 0.182), indicating that weaker confounding could attenuate this association.

The results do not provide evidence that residential mobility significantly influences burglary. The association remains close to zero across both the simple and fixed‐effects models. One explanation relates to the way burglary is measured in police‐recorded data, which combine residential and commercial burglary within the same category. These offences involve different opportunity structures and situational conditions. Residential burglary risk is shaped by occupancy patterns, neighbourhood guardianship, and routine activities of residents (Clarke & Felson, 1993; Tilley et al., 2015; Wilcox et al., 2007). In contrast, commercial burglary is more closely linked to business density, security measures, and the absence of on-site supervision (Butler, 2005; Ekblom, 2014; Walsh, 2019). In this context, the absence of a measurable relationship does not necessarily indicate a lack of effect but rather the cancellation of two contrasting trends aggregated within burglary crime category.

The results support H2, indicating that increases in transit station mobility are associated with higher levels of theft from the person. The association is consistent with prior research linking crowding in public transport systems to greater opportunities for pickpocketing and similar offences (Ceccato & Uittenbogaard, 2014; Newton, 2014; Smith & Clarke, 2000). Changes in commuting patterns have also been shown to significant impact on crime outcomes (Mburu & Helbich, 2016). Such environments concentrate large numbers of transient individuals carrying personal belongings, creating conditions in which offenders can approach suitable targets with limited risk of detection. In contrast, the simple model displays opposite relationships. Introducing area fixed effects reverses these patterns, revealing that much of the apparent cross-sectional variation in the simple models stems from time-invariant between-area differences rather than within-area dynamics. Once unobserved local characteristics are controlled for, transit mobility emerges as a predictor of theft person incidents.

When mobility increases, we may also expect guardianship to rise, since more people are present who could potentially observe and report suspicious behaviour. This apparent paradox has been addressed in the Guardianship in Action framework (Reynald, 2009, 2010), which emphasises that mere physical presence does not automatically translate into active guardianship. Guardians must be willing and able to intervene or signal awareness of potential offences. High-density environments such as transport hubs may contain many bystanders but limited active guardianship, resulting in low effective guardianship despite high visibility (Hollis-Peel & Welsh, 2014; Hollis-Peel et al., 2011, 2012). The positive mobility-theft relationship, therefore, does not contradict guardianship theory but rather illustrates the difference between passive presence and active surveillance.

Contrary to H3, workplace mobility is not the strongest predictor of violence and sexual offences. Although the models indicate a positive association, this relationship is weaker compared to retail and grocery mobility. This result should be interpreted cautiously because the Home Office category combines a wide range of offences, including assaults, bodily harm, and sexual offences, which may respond differently to mobility patterns. The aggregated measure therefore does not isolate a single mechanism or setting of violence.

Evidence from occupational safety and harassment studies shows that sexual harassment and assault can occur in workplace settings, particularly in healthcare, education, and hospitality sectors (McDonald, 2012; Willness et al., 2007). One possible explanation is related to the reorganisation of workplaces during the pandemic, as many employees worked remotely or under hybrid arrangements. This shift may have reduced physical interaction in traditional workplaces, while essential shops and services remained open, concentrating interpersonal contact in these locations. Additional evidence supports the idea that violence concentrated in retail and essential-service locations during the pandemic. Bushell and Braithwaite (2024); Taylor (2022) found increases in verbal abuse, threats, and assaults against retail workers during and after lockdown periods. The stronger association for retail-related mobility may therefore reflect both the concentration of contact in essential-service settings and the broad composition of the violence and sexual offences category.

The results provide clear support for the final hypothesis that retail and recreation mobility are positively related to shoplifting and theft from the person, whereas robbery shows a much weaker association. This finding aligns with previous research linking high pedestrian density and commercial activity to increases in low-risk acquisitive offences (Gill, 2007; Reid et al., 2021). In contrast, robbery entails direct confrontation and higher risk of apprehension, which makes it less sensitive to fluctuations in retail footfall. Empirical research confirms that robbery risk is associated with situational factors such as time of day, the presence of weapons, and environmental isolation rather than simple increases in public activity (Wright & Decker, 1997).

Residential mobility shows a negative association with almost all crime categories, except for drug offences and ASB (Fig. 3). During lockdowns, much of what was recorded as ASB related to breaches of public health restrictions, neighbour disputes, or minor disturbances arising from prolonged confinement. These incidents inflated ASB counts in residential areas while other forms of crime declined, producing the opposite pattern seen for most other offence types. Drug offences follow a similar pattern, though generally smaller effects were found. Higher residential mobility showed a positive association, suggesting proactive policing (Langton et al., 2021b).

The negative direction indicates that when more time is spent in or near residential areas, crime levels generally decline. This pattern likely reflects a broader contraction of routine activities during periods of restricted mobility. When people remain closer to home, both potential offenders and potential targets circulate less in public activity spaces, reducing opportunities for many acquisitive and public-space offences. These results should also be interpreted with caution because several Home Office offence groups aggregate different behaviours that may respond differently to mobility changes. For example, categories such as violence and sexual offences, other theft, and vehicle crime combine incidents occurring in diverse settings and involving distinct opportunity structures. As a result, the estimated associations capture the average response across heterogeneous offence types rather than a single underlying mechanism.

It should be acknowledged that some of the patterns discussed above likely reflect mechanisms that extend beyond the opportunity-based logic of Routine Activity Theory. Changes in enforcement practices, such as the shift toward proactive policing during lockdowns, may account for part of the pattern observed for drug offences independently of changes in ambient population. Similarly, the concentration of violence in parks and public spaces during later pandemic phases may partly reflect accumulated social tensions and deteriorating informal social controls following prolonged confinement, rather than a straightforward increase in suitable targets and offenders converging in space. RAT provides a coherent framework for understanding how changes in routine activities alter crime opportunities, but the pandemic created conditions in which economic strain, enforcement reorientation, and institutional reorganisation operated simultaneously. These factors are beyond the scope of the present empirical analysis, but situating the findings within this broader context clarifies what RAT can reasonably account for and where its explanatory reach is limited.

This study has several limitations related to the use of police-recorded crime data and Google Community Mobility Reports. Police data capture only offences that are reported and recorded, meaning that the results reflect trends in recorded incidents rather than total crime. Reporting behaviour and policing practices may also have changed during the pandemic; and these may vary across offence type. To assess the potential influence of confounders, we conducted sensitivity analyses using the robustness value approach. The estimated robustness values suggest that even the weakest associations would require an unobserved confounder to explain more than 18% of the residual variation in both the mobility indicator and the corresponding offence type to fully eliminate the observed effect. We argue that this is an unrealistically high threshold. Let us take for example, law enforcement, and, for the sake of the argument, let us assume that this unobserved variable acts entirely as a confounder and not a mediator—which could be the case if police activity was to respond to changing crime rates (Kim et al., 2024)—and that no differential measurement error is present—i.e. crime rates reflect the true extent of criminal activity and not how effective the police is at detecting crime (Pina-Sánchez et al., 2023). Changes in police presence should be the cause behind 18% of the change in mobility and crime. The latter cannot be ruled out, even if the evidence in support of the effectiveness of police presence in reducing crime beyond hotspots is not that clear. The former however is completely unrealistic, police presence at the LSOA level cannot be expected to predict about a sixth of mobility around retail areas.

Limitations also arise from the measurement of mobility. GMR data represent only users who enable location history on their Google accounts and may therefore not perfectly represent the entire population. However, such bias would affect the estimates only if the composition of users changed substantially during the study period. If the composition is relatively stable within areas, any resulting measurement error is time in variant and absorbed by the fixed effects, limiting its impact on the estimated relationships. Missing observations were addressed through multiple imputation using temporal and spatial covariates, although this approach relies on a missing-at-random assumption. The parks category contains higher levels of missingness, likely reflecting lower user density in these spaces, and the corresponding estimates should therefore be interpreted with greater caution. In addition, the January–February 2020 baseline used by Google represents a short pre-pandemic period rather than a full seasonal cycle, which may affect the interpretation of percentage changes but is unlikely to alter the relative relationships between mobility categories. A further limitation concerns temporal aggregation. Crime data are available only at monthly intervals for local authorities, which prevents examination of short-term sequencing between mobility and crime within the same month. The models therefore estimate associations between monthly changes in mobility and crime rather than identifying precise temporal ordering. Although the longitudinal panel and fixed-effects design reduce bias from stable local characteristics, some temporal aggregation bias may remain.

## Conclusion

The findings show that pandemic-related mobility changes were systematically associated with police-recorded crime across England and Wales, but the strength of these associations varied by offence type and activity space. Greater residential mobility was generally associated with lower crime, whereas increased mobility in retail, recreation, and transit settings was more often associated with higher crime. The strongest and most consistent relationships were observed for theft from the person and shoplifting, while burglary and violence and sexual offences were more difficult to interpret because the available offence groups combine incidents with different situational dynamics. Overall, the results indicate that mobility matters not as a generic measure of movement, but as an indicator of where offender-target convergence and guardianship are concentrated.

Future research should extend this analysis in three specific ways. First, it should test whether similar mobility-crime relationships persist beyond the pandemic by using post-pandemic time series and alternative shocks to routine activity, such as major transport disruptions, economic closures, or changes in working patterns. Second, it should use more disaggregated offence data, particularly separating residential from commercial burglary and, where possible, disaggregating violence and sexual offences, to examine whether mobility operates differently across offence subtypes currently grouped together in police data. Third, daily or weekly crime data linked to higher-frequency mobility measures with alternative mobility indicators, such as transport usage, footfall sensors, or mobile-phone mobility traces, which would allow closer examination of short-term temporal ordering and underlying mechanisms.

The policy implications are similarly offence- and place-specific. The results suggest that prevention efforts should be directed towards the activity spaces most closely associated with each offence type rather than applied uniformly across areas. For example, where retail and transit mobility are strongly linked to theft from the person or shoplifting, targeted surveillance, visible guardianship, staff presence, and situational prevention in shopping centres, transport hubs, and surrounding corridors are likely to be more effective than area-wide enforcement. Where public order and violence are more closely tied to parks or mixed-use leisure settings, prevention may require place management, staffing, lighting, and crowd monitoring rather than conventional police deployment alone. More broadly, policies should be sensitive to the structural and social characteristics of areas, including land use, transport infrastructure, commercial density, and patterns of public congregation, because the same change in mobility may generate different crime risks in different local contexts.

## Supplementary Information

Additional file1 (DOCX 1015 kb)

## Data Availability

The crime data and mobility data used in this study are publicly available and retrievable. The crime data was obtained from https://data.police.uk/data/. The mobility data, on the other hand, was obtained from Google Mobility Reports. The code files for the analysis available https://github.com/hulyas000/Mobility_and_crime_paper

## Footnotes

1 Retrieved from data.police.uk.

2 Lookup file can be found: https://www.data.gov.uk/dataset/8b689993-7ffa-46ff-a0d8-5186d2deafd9/lsoa-2011-to-electoral-ward-2019-to-lad-2019-best-fit-lookup-in-ew1

3 According to April 2019 administrative unites. See for more information on local government structure: https://www.gov.uk/guidance/local-government-structure-and-elections#structure, accessed on 20 March 2025.

4 The Office of National Statics’ establishment to find new sources of data. The mapping file for disparities in Google places and UK boundaries can be found: (https://github.com/datasciencecampus/google-mobility-reports-data/tree/master/geography).

5 Plots illustrating these distributions are provided in the Online Appendix Figs. 1A-3A.

6 We also specified simpler Poisson models, but the negative binomial models offered a better fit throughout.

7 Models including month fixed effects are reported in the Appendix – Table 1A as robustness checks. Standard errors are clustered at the LAD level to account for serial correlation.

8 The results are given in the next section in detail.
