# Supplementary material for: The association between mobility and crime in England and Wales
Source: Crime Sci. 2026 Jul 15;15(1):22. doi: 10.1186/s40163-026-00291-z (PMC13372996; doi:10.1186/s40163-026-00291-z)
Supplement: Supplementary file 1 — Additional file1 (DOCX 1015 kb) [file 40163_2026_291_MOESM1_ESM.docx]

Appendix


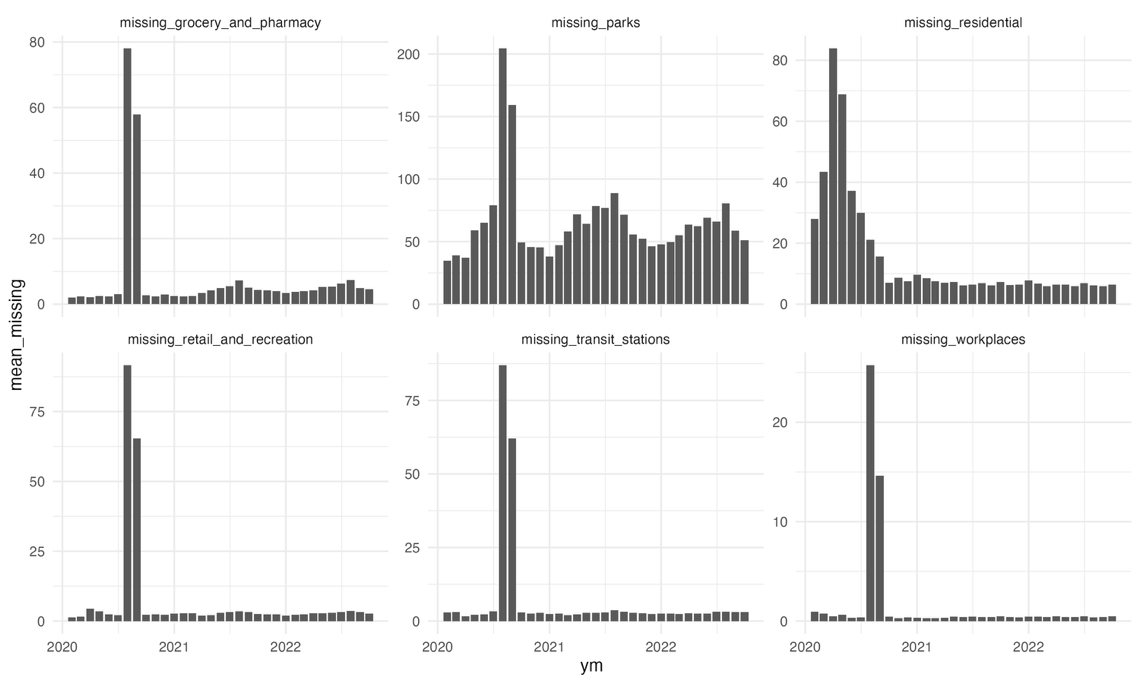


**Figure 1A: Temporal distribution of missing mobility observations by calendar month during the study period.**

The figure above shows the mean proportion of missing values in Google Community Mobility indicators across months between 2020 and 2022 for each mobility category. Missingness is concentrated in specific periods, particularly mid-2020, indicating that missing mobility data are unevenly distributed over time.


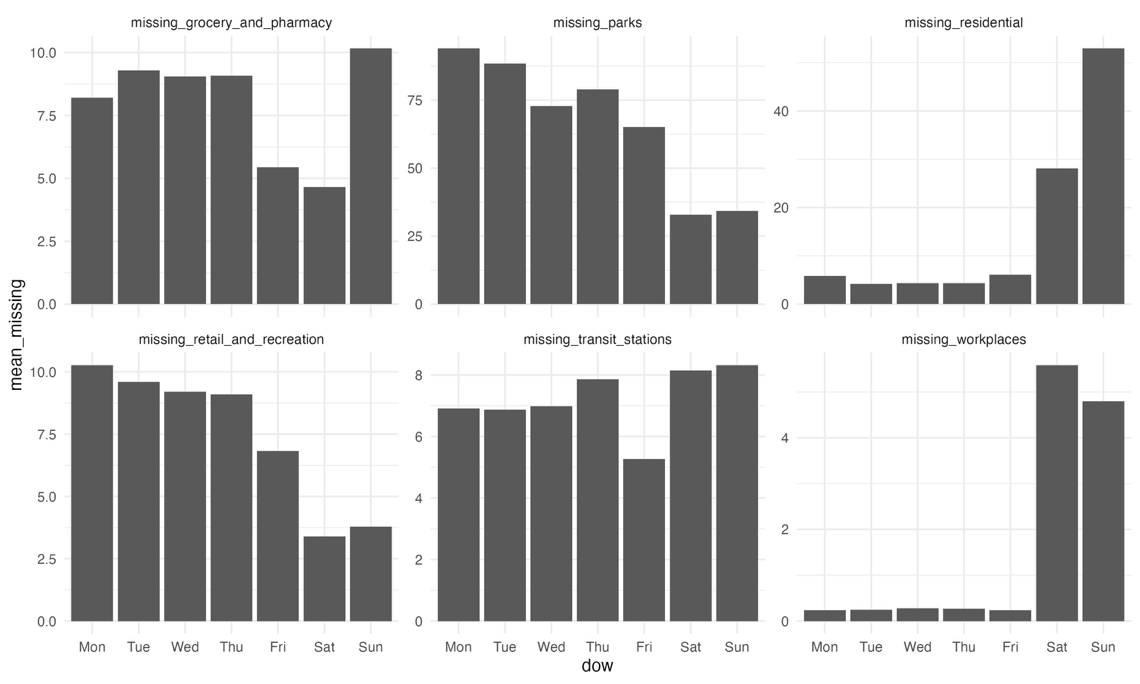


**Figure 2A: Distribution of missing mobility observations by day of the week.**

The figure reports the mean proportion of missing values across mobility indicators for each day of the week. Missingness is systematically higher on weekends for several mobility streams, indicating a non-random temporal pattern in the availability of mobility data.


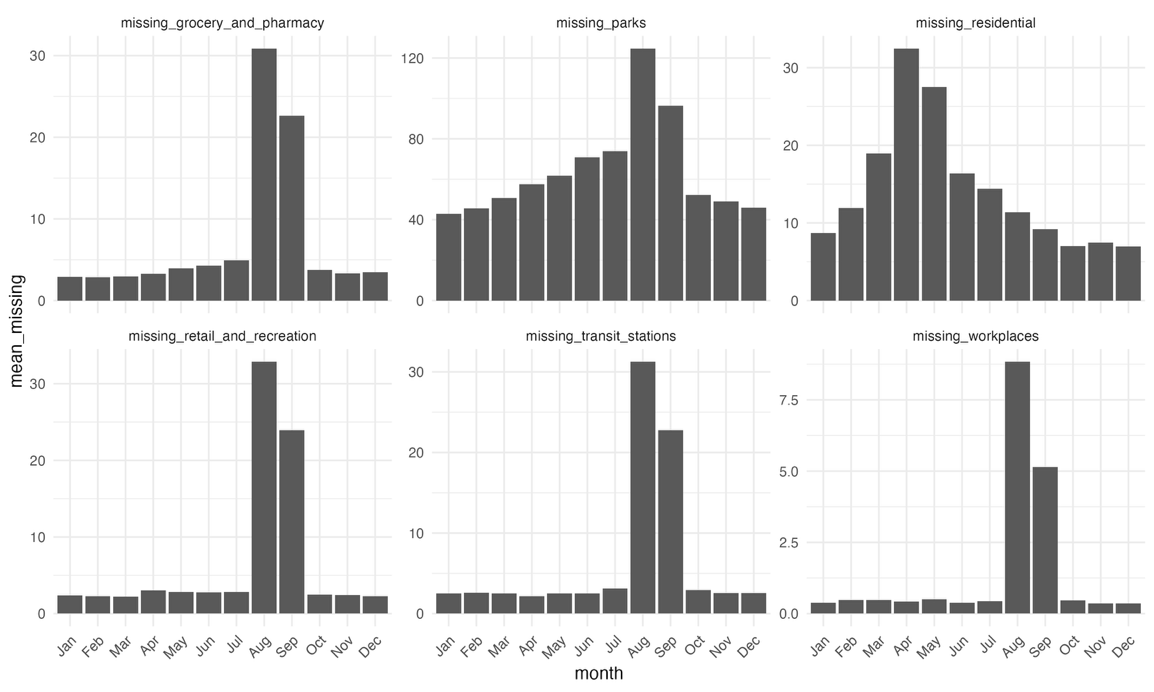


**Figure 3A: Seasonal distribution of missing mobility observations by month of the year.**

The figure presents the mean proportion of missing values by calendar month aggregated across years. Missing mobility observations are unevenly distributed across the annual cycle, with notable concentrations during late summer months. These temporal patterns were considered when implementing multiple imputation for mobility indicators.

The heatmaps below present standardised beta coefficients from area fixed-effects and simple models including time fixed effects for each offence category and mobility indicator. Colours represent the magnitude and direction of the association, with warmer colours indicating positive relationships and cooler colours indicating negative relationships. Cells marked with asterisks denote statistically significant coefficients. Greyed rows indicate offence categories for which models could not be estimated due to insufficient variation. The figure allows comparison with the fixed-effects specification presented in the main analysis, illustrating how associations between mobility indicators and crime outcomes differ when time-variant factors are controlled.


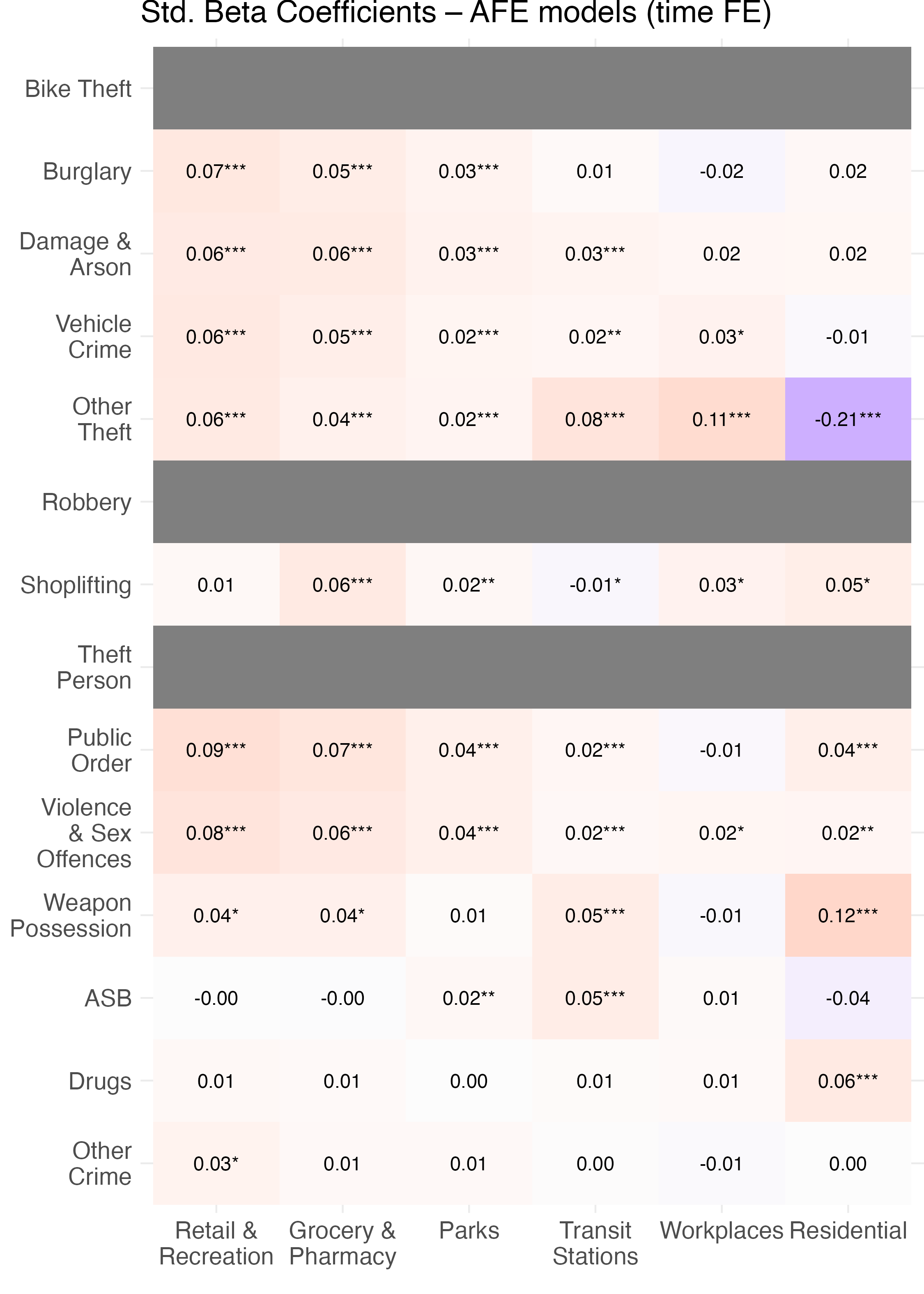


**Figure 4A: Std. beta coefficients for mobility indicators in area fixed-effects models with time fixed effects.**


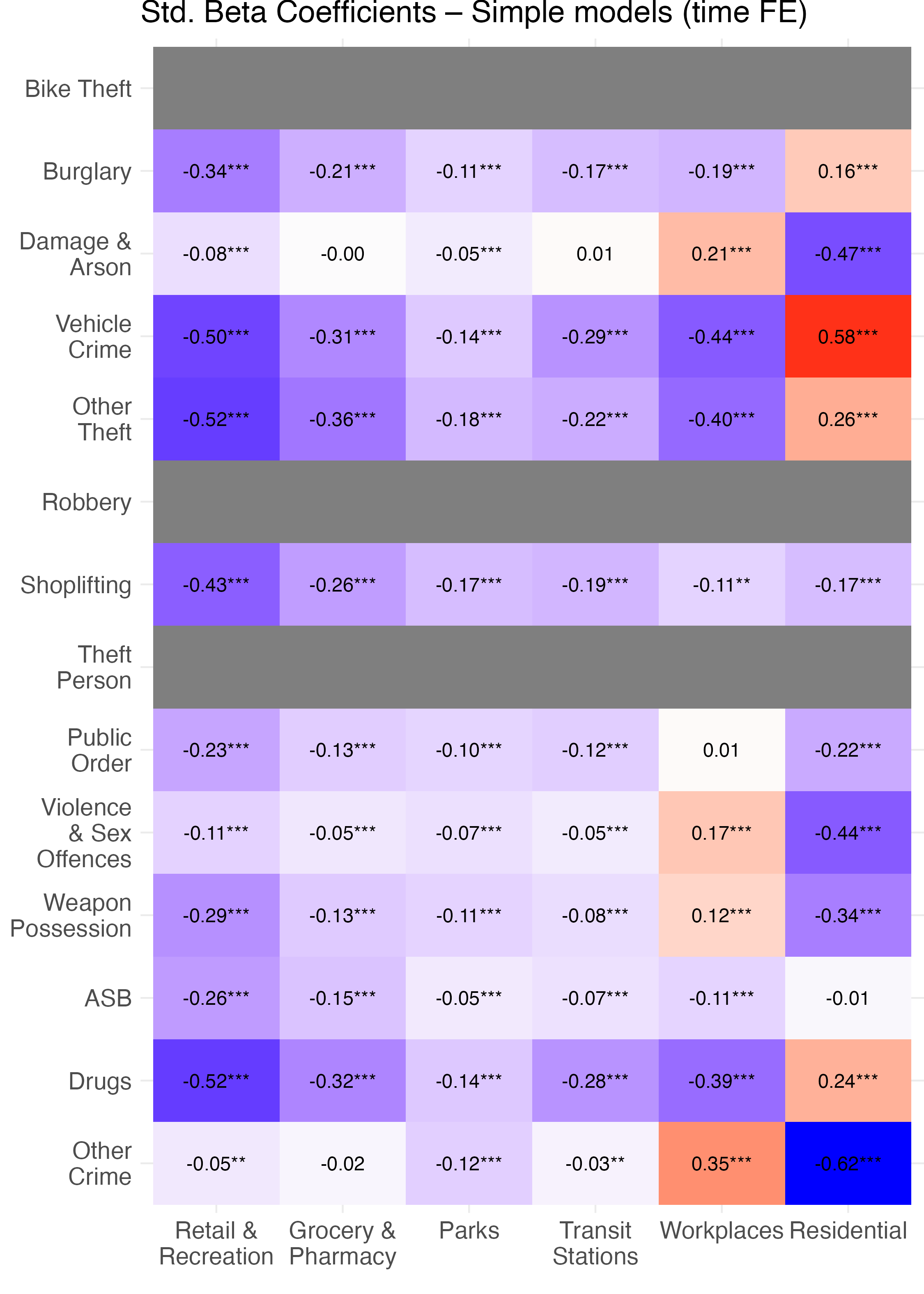


**Figure 5A: Std. beta coefficients for mobility indicators in simple models with time fixed effects**.

The table reports coefficient estimates, standard errors, confidence intervals, p-values, pseudo-R², partial R², and sample sizes for all model specifications, including simple and area fixed-effects models with and without time fixed effects. Mobility measures correspond to Google Community Mobility categories

**Table 1A: Full regression results for mobility–crime models across offence types and mobility categories in England and Wales.**

| **Crime Type** | **Mobility** | **Model** | **Spec** | **Intercept** | **intercept Se** | **Estimate** | **Estimate SE** | **CI Low** | **CI High** | **P value** | **Pseudo R2** | **Partial R2** | **N** | **DF Residual** |
| --- | --- | --- | --- | --- | --- | --- | --- | --- | --- | --- | --- | --- | --- | --- |
| ASB | retail and recreation | afe | noTimeFE | -5.827 | 0.040 | -0.009 | 0.000 | -0.009 | -0.009 | 0.000 | 0.75 | 0.20 | 10432 | 10105 |
| ASB | retail and recreation | simple | noTimeFE | -6.436 | 0.011 | -0.010 | 0.000 | -0.011 | -0.009 | 0.000 | 0.18 | 0.05 | 10432 | 10430 |
| ASB | retail and recreation | afe | timeFE | -5.643 | 0.028 | 0.000 | 0.000 | -0.001 | 0.001 | 0.982 | 0.86 | 0.00 | 10432 | 10074 |
| ASB | retail and recreation | simple | timeFE | -6.537 | 0.037 | -0.009 | 0.001 | -0.011 | -0.007 | 0.000 | 0.28 | 0.01 | 10432 | 10399 |
| ASB | grocery and pharmacy | afe | noTimeFE | -5.612 | 0.039 | -0.015 | 0.000 | -0.016 | -0.014 | 0.000 | 0.74 | 0.15 | 10432 | 10105 |
| ASB | grocery and pharmacy | simple | noTimeFE | -6.228 | 0.006 | -0.013 | 0.001 | -0.015 | -0.012 | 0.000 | 0.14 | 0.04 | 10432 | 10430 |
| ASB | grocery and pharmacy | afe | timeFE | -5.643 | 0.026 | 0.000 | 0.000 | -0.001 | 0.001 | 0.705 | 0.86 | 0.00 | 10432 | 10074 |
| ASB | grocery and pharmacy | simple | timeFE | -6.318 | 0.028 | -0.009 | 0.001 | -0.010 | -0.007 | 0.000 | 0.26 | 0.01 | 10432 | 10399 |
| ASB | parks | afe | noTimeFE | -5.552 | 0.063 | 0.000 | 0.000 | 0.000 | 0.000 | 0.118 | 0.64 | 0.00 | 10342 | 10015 |
| ASB | parks | simple | noTimeFE | -6.168 | 0.009 | -0.001 | 0.000 | -0.001 | -0.001 | 0.000 | 0.00 | 0.00 | 10342 | 10340 |
| ASB | parks | afe | timeFE | -5.627 | 0.026 | 0.000 | 0.000 | 0.000 | 0.001 | 0.002 | 0.86 | 0.00 | 10342 | 9984 |
| ASB | parks | simple | timeFE | -6.297 | 0.029 | -0.001 | 0.000 | -0.001 | -0.001 | 0.000 | 0.24 | 0.00 | 10342 | 10309 |
| ASB | transit stations | afe | noTimeFE | -5.750 | 0.055 | -0.007 | 0.000 | -0.008 | -0.007 | 0.000 | 0.68 | 0.08 | 10432 | 10105 |
| ASB | transit stations | simple | noTimeFE | -6.414 | 0.013 | -0.007 | 0.000 | -0.008 | -0.006 | 0.000 | 0.07 | 0.03 | 10432 | 10430 |
| ASB | transit stations | afe | timeFE | -5.603 | 0.026 | 0.002 | 0.000 | 0.002 | 0.002 | 0.000 | 0.86 | 0.01 | 10432 | 10074 |
| ASB | transit stations | simple | timeFE | -6.368 | 0.030 | -0.003 | 0.000 | -0.004 | -0.002 | 0.000 | 0.24 | 0.01 | 10432 | 10399 |
| ASB | workplaces | afe | noTimeFE | -6.049 | 0.036 | -0.021 | 0.000 | -0.021 | -0.020 | 0.000 | 0.77 | 0.33 | 10432 | 10105 |
| ASB | workplaces | simple | noTimeFE | -6.756 | 0.019 | -0.018 | 0.001 | -0.019 | -0.016 | 0.000 | 0.14 | 0.08 | 10432 | 10430 |
| ASB | workplaces | afe | timeFE | -5.633 | 0.028 | 0.001 | 0.001 | -0.001 | 0.002 | 0.417 | 0.86 | 0.00 | 10432 | 10074 |
| ASB | workplaces | simple | timeFE | -6.465 | 0.039 | -0.008 | 0.001 | -0.010 | -0.005 | 0.000 | 0.24 | 0.00 | 10432 | 10399 |
| ASB | residential | afe | noTimeFE | -5.854 | 0.055 | 0.042 | 0.001 | 0.041 | 0.043 | 0.000 | 0.76 | 0.33 | 10395 | 10068 |
| ASB | residential | simple | noTimeFE | -6.578 | 0.012 | 0.038 | 0.001 | 0.036 | 0.040 | 0.000 | 0.13 | 0.10 | 10395 | 10393 |
| ASB | residential | afe | timeFE | -5.604 | 0.032 | -0.006 | 0.003 | -0.012 | 0.000 | 0.038 | 0.86 | 0.00 | 10395 | 10037 |
| ASB | residential | simple | timeFE | -6.283 | 0.041 | -0.002 | 0.003 | -0.009 | 0.004 | 0.500 | 0.24 | 0.00 | 10395 | 10362 |
| Bike Theft | retail and recreation | afe | noTimeFE | -9.098 | 0.078 | 0.005 | 0.000 | 0.004 | 0.005 | 0.000 | 0.80 | 0.05 | 9898 | 9571 |
| Bike Theft | retail and recreation | simple | noTimeFE | -9.427 | 0.015 | -0.011 | 0.001 | -0.012 | -0.009 | 0.000 | 0.06 | 0.02 | 9898 | 9896 |
| Bike Theft | retail and recreation | afe | timeFE |  |  |  |  |  |  |  |  |  |  |  |
| Bike Theft | retail and recreation | simple | timeFE |  |  |  |  |  |  |  |  |  |  |  |
| Bike Theft | grocery and pharmacy | afe | noTimeFE | -9.217 | 0.079 | 0.007 | 0.000 | 0.006 | 0.008 | 0.000 | 0.80 | 0.03 | 9898 | 9571 |
| Bike Theft | grocery and pharmacy | simple | noTimeFE | -9.219 | 0.013 | -0.018 | 0.002 | -0.021 | -0.015 | 0.000 | 0.09 | 0.01 | 9898 | 9896 |
| Bike Theft | grocery and pharmacy | afe | timeFE |  |  |  |  |  |  |  |  |  |  |  |
| Bike Theft | grocery and pharmacy | simple | timeFE |  |  |  |  |  |  |  |  |  |  |  |
| Bike Theft | parks | afe | noTimeFE | -9.109 | 0.083 | 0.004 | 0.000 | 0.004 | 0.004 | 0.000 | 0.81 | 0.07 | 9811 | 9484 |
| Bike Theft | parks | simple | noTimeFE | -9.086 | 0.025 | -0.003 | 0.001 | -0.003 | -0.002 | 0.000 | 0.01 | 0.00 | 9811 | 9809 |
| Bike Theft | parks | afe | timeFE |  |  |  |  |  |  |  |  |  |  |  |
| Bike Theft | parks | simple | timeFE |  |  |  |  |  |  |  |  |  |  |  |
| Bike Theft | transit stations | afe | noTimeFE | -9.043 | 0.082 | 0.007 | 0.000 | 0.007 | 0.008 | 0.000 | 0.80 | 0.06 | 9898 | 9571 |
| Bike Theft | transit stations | simple | noTimeFE | -9.543 | 0.022 | -0.011 | 0.001 | -0.013 | -0.010 | 0.000 | 0.06 | 0.02 | 9898 | 9896 |
| Bike Theft | transit stations | afe | timeFE |  |  |  |  |  |  |  |  |  |  |  |
| Bike Theft | transit stations | simple | timeFE |  |  |  |  |  |  |  |  |  |  |  |
| Bike Theft | workplaces | afe | noTimeFE | -9.080 | 0.080 | 0.007 | 0.001 | 0.006 | 0.008 | 0.000 | 0.79 | 0.02 | 9898 | 9571 |
| Bike Theft | workplaces | simple | noTimeFE | -9.878 | 0.041 | -0.022 | 0.002 | -0.025 | -0.019 | 0.000 | 0.07 | 0.02 | 9898 | 9896 |
| Bike Theft | workplaces | afe | timeFE |  |  |  |  |  |  |  |  |  |  |  |
| Bike Theft | workplaces | simple | timeFE |  |  |  |  |  |  |  |  |  |  |  |
| Bike Theft | residential | afe | noTimeFE | -9.086 | 0.080 | -0.021 | 0.001 | -0.023 | -0.019 | 0.000 | 0.79 | 0.05 | 9863 | 9536 |
| Bike Theft | residential | simple | noTimeFE | -9.326 | 0.023 | 0.012 | 0.002 | 0.008 | 0.017 | 0.000 | 0.01 | 0.00 | 9863 | 9861 |
| Bike Theft | residential | afe | timeFE |  |  |  |  |  |  |  |  |  |  |  |
| Bike Theft | residential | simple | timeFE |  |  |  |  |  |  |  |  |  |  |  |
| Burglary | retail and recreation | afe | noTimeFE | -7.225 | 0.040 | 0.001 | 0.000 | 0.001 | 0.001 | 0.000 | 0.73 | 0.01 | 10431 | 10104 |
| Burglary | retail and recreation | simple | noTimeFE | -8.098 | 0.007 | -0.003 | 0.000 | -0.003 | -0.003 | 0.000 | 0.02 | 0.02 | 10431 | 10429 |
| Burglary | retail and recreation | afe | timeFE | -6.925 | 0.042 | 0.002 | 0.000 | 0.002 | 0.003 | 0.000 | 0.76 | 0.00 | 10431 | 10073 |
| Burglary | retail and recreation | simple | timeFE | -8.089 | 0.042 | -0.011 | 0.001 | -0.014 | -0.009 | 0.000 | 0.14 | 0.01 | 10431 | 10398 |
| Burglary | grocery and pharmacy | afe | noTimeFE | -7.249 | 0.040 | 0.001 | 0.000 | 0.000 | 0.001 | 0.001 | 0.73 | 0.00 | 10431 | 10104 |
| Burglary | grocery and pharmacy | simple | noTimeFE | -8.040 | 0.005 | -0.006 | 0.001 | -0.007 | -0.005 | 0.000 | 0.03 | 0.01 | 10431 | 10429 |
| Burglary | grocery and pharmacy | afe | timeFE | -6.986 | 0.040 | 0.003 | 0.000 | 0.002 | 0.004 | 0.000 | 0.76 | 0.00 | 10431 | 10073 |
| Burglary | grocery and pharmacy | simple | timeFE | -7.812 | 0.026 | -0.011 | 0.001 | -0.013 | -0.009 | 0.000 | 0.10 | 0.01 | 10431 | 10398 |
| Burglary | parks | afe | noTimeFE | -7.253 | 0.041 | 0.000 | 0.000 | 0.000 | 0.000 | 0.107 | 0.73 | 0.00 | 10341 | 10014 |
| Burglary | parks | simple | noTimeFE | -7.981 | 0.007 | -0.002 | 0.000 | -0.002 | -0.001 | 0.000 | 0.03 | 0.01 | 10341 | 10339 |
| Burglary | parks | afe | timeFE | -6.963 | 0.041 | 0.001 | 0.000 | 0.000 | 0.001 | 0.000 | 0.77 | 0.00 | 10341 | 9983 |
| Burglary | parks | simple | timeFE | -7.787 | 0.027 | -0.002 | 0.000 | -0.002 | -0.002 | 0.000 | 0.06 | 0.01 | 10341 | 10308 |
| Burglary | transit stations | afe | noTimeFE | -7.218 | 0.040 | 0.001 | 0.000 | 0.001 | 0.002 | 0.000 | 0.73 | 0.01 | 10431 | 10104 |
| Burglary | transit stations | simple | noTimeFE | -8.140 | 0.008 | -0.003 | 0.000 | -0.004 | -0.003 | 0.000 | 0.02 | 0.02 | 10431 | 10429 |
| Burglary | transit stations | afe | timeFE | -6.986 | 0.040 | 0.000 | 0.000 | 0.000 | 0.001 | 0.096 | 0.76 | 0.00 | 10431 | 10073 |
| Burglary | transit stations | simple | timeFE | -7.950 | 0.027 | -0.006 | 0.000 | -0.007 | -0.006 | 0.000 | 0.08 | 0.03 | 10431 | 10398 |
| Burglary | workplaces | afe | noTimeFE | -7.196 | 0.040 | 0.003 | 0.000 | 0.002 | 0.003 | 0.000 | 0.73 | 0.01 | 10431 | 10104 |
| Burglary | workplaces | simple | noTimeFE | -8.113 | 0.015 | -0.003 | 0.001 | -0.004 | -0.002 | 0.000 | 0.00 | 0.00 | 10431 | 10429 |
| Burglary | workplaces | afe | timeFE | -7.011 | 0.041 | -0.001 | 0.001 | -0.003 | 0.001 | 0.195 | 0.76 | 0.00 | 10431 | 10073 |
| Burglary | workplaces | simple | timeFE | -8.076 | 0.035 | -0.013 | 0.001 | -0.015 | -0.011 | 0.000 | 0.07 | 0.01 | 10431 | 10398 |
| Burglary | residential | afe | noTimeFE | -7.228 | 0.040 | -0.003 | 0.001 | -0.004 | -0.003 | 0.000 | 0.72 | 0.01 | 10394 | 10067 |
| Burglary | residential | simple | noTimeFE | -8.049 | 0.010 | 0.001 | 0.001 | -0.001 | 0.003 | 0.295 | 0.00 | 0.00 | 10394 | 10392 |
| Burglary | residential | afe | timeFE | -7.010 | 0.041 | 0.002 | 0.002 | -0.002 | 0.006 | 0.266 | 0.76 | 0.00 | 10394 | 10036 |
| Burglary | residential | simple | timeFE | -8.018 | 0.036 | 0.025 | 0.003 | 0.019 | 0.030 | 0.000 | 0.05 | 0.01 | 10394 | 10361 |
| Damage & Arson | retail and recreation | afe | noTimeFE | -6.518 | 0.028 | 0.003 | 0.000 | 0.003 | 0.003 | 0.000 | 0.83 | 0.15 | 10432 | 10105 |
| Damage & Arson | retail and recreation | simple | noTimeFE | -7.280 | 0.005 | 0.002 | 0.000 | 0.002 | 0.002 | 0.000 | 0.01 | 0.01 | 10432 | 10430 |
| Damage & Arson | retail and recreation | afe | timeFE | -6.534 | 0.027 | 0.002 | 0.000 | 0.001 | 0.002 | 0.000 | 0.86 | 0.01 | 10432 | 10074 |
| Damage & Arson | retail and recreation | simple | timeFE | -7.370 | 0.026 | -0.002 | 0.000 | -0.003 | -0.001 | 0.000 | 0.06 | 0.00 | 10432 | 10399 |
| Damage & Arson | grocery and pharmacy | afe | noTimeFE | -6.595 | 0.027 | 0.005 | 0.000 | 0.005 | 0.006 | 0.000 | 0.83 | 0.12 | 10432 | 10105 |
| Damage & Arson | grocery and pharmacy | simple | noTimeFE | -7.321 | 0.004 | 0.003 | 0.000 | 0.003 | 0.004 | 0.000 | 0.01 | 0.01 | 10432 | 10430 |
| Damage & Arson | grocery and pharmacy | afe | timeFE | -6.582 | 0.026 | 0.003 | 0.000 | 0.002 | 0.003 | 0.000 | 0.86 | 0.01 | 10432 | 10074 |
| Damage & Arson | grocery and pharmacy | simple | timeFE | -7.311 | 0.023 | 0.000 | 0.001 | -0.001 | 0.001 | 0.806 | 0.05 | 0.00 | 10432 | 10399 |
| Damage & Arson | parks | afe | noTimeFE | -6.561 | 0.028 | 0.002 | 0.000 | 0.001 | 0.002 | 0.000 | 0.82 | 0.08 | 10342 | 10015 |
| Damage & Arson | parks | simple | noTimeFE | -7.327 | 0.006 | 0.000 | 0.000 | 0.000 | 0.000 | 0.217 | 0.00 | 0.00 | 10342 | 10340 |
| Damage & Arson | parks | afe | timeFE | -6.557 | 0.027 | 0.001 | 0.000 | 0.000 | 0.001 | 0.000 | 0.86 | 0.01 | 10342 | 9984 |
| Damage & Arson | parks | simple | timeFE | -7.310 | 0.023 | -0.001 | 0.000 | -0.001 | 0.000 | 0.000 | 0.06 | 0.00 | 10342 | 10309 |
| Damage & Arson | transit stations | afe | noTimeFE | -6.503 | 0.028 | 0.004 | 0.000 | 0.004 | 0.004 | 0.000 | 0.83 | 0.12 | 10432 | 10105 |
| Damage & Arson | transit stations | simple | noTimeFE | -7.250 | 0.007 | 0.002 | 0.000 | 0.002 | 0.003 | 0.000 | 0.01 | 0.02 | 10432 | 10430 |
| Damage & Arson | transit stations | afe | timeFE | -6.571 | 0.027 | 0.001 | 0.000 | 0.001 | 0.001 | 0.000 | 0.86 | 0.00 | 10432 | 10074 |
| Damage & Arson | transit stations | simple | timeFE | -7.306 | 0.024 | 0.000 | 0.000 | 0.000 | 0.001 | 0.454 | 0.05 | 0.00 | 10432 | 10399 |
| Damage & Arson | workplaces | afe | noTimeFE | -6.472 | 0.029 | 0.006 | 0.000 | 0.006 | 0.006 | 0.000 | 0.83 | 0.12 | 10432 | 10105 |
| Damage & Arson | workplaces | simple | noTimeFE | -7.065 | 0.011 | 0.009 | 0.000 | 0.008 | 0.009 | 0.000 | 0.06 | 0.06 | 10432 | 10430 |
| Damage & Arson | workplaces | afe | timeFE | -6.574 | 0.028 | 0.001 | 0.001 | 0.000 | 0.002 | 0.055 | 0.86 | 0.00 | 10432 | 10074 |
| Damage & Arson | workplaces | simple | timeFE | -7.032 | 0.028 | 0.013 | 0.001 | 0.011 | 0.014 | 0.000 | 0.10 | 0.03 | 10432 | 10399 |
| Damage & Arson | residential | afe | noTimeFE | -6.515 | 0.027 | -0.013 | 0.000 | -0.014 | -0.013 | 0.000 | 0.83 | 0.15 | 10395 | 10068 |
| Damage & Arson | residential | simple | noTimeFE | -7.129 | 0.008 | -0.022 | 0.001 | -0.023 | -0.021 | 0.000 | 0.09 | 0.10 | 10395 | 10393 |
| Damage & Arson | residential | afe | timeFE | -6.606 | 0.030 | 0.002 | 0.002 | -0.001 | 0.006 | 0.161 | 0.86 | 0.00 | 10395 | 10037 |
| Damage & Arson | residential | simple | timeFE | -6.759 | 0.027 | -0.063 | 0.002 | -0.066 | -0.059 | 0.000 | 0.16 | 0.10 | 10395 | 10362 |
| Drugs | retail and recreation | afe | noTimeFE | -8.030 | 0.067 | -0.004 | 0.000 | -0.005 | -0.004 | 0.000 | 0.81 | 0.09 | 10419 | 10092 |
| Drugs | retail and recreation | simple | noTimeFE | -8.619 | 0.010 | -0.010 | 0.000 | -0.010 | -0.009 | 0.000 | 0.13 | 0.05 | 10419 | 10417 |
| Drugs | retail and recreation | afe | timeFE | -7.925 | 0.062 | 0.001 | 0.000 | 0.000 | 0.001 | 0.204 | 0.82 | 0.00 | 10419 | 10061 |
| Drugs | retail and recreation | simple | timeFE | -8.948 | 0.061 | -0.019 | 0.002 | -0.023 | -0.015 | 0.000 | 0.20 | 0.01 | 10419 | 10386 |
| Drugs | grocery and pharmacy | afe | noTimeFE | -7.924 | 0.064 | -0.007 | 0.000 | -0.007 | -0.006 | 0.000 | 0.80 | 0.06 | 10419 | 10092 |
| Drugs | grocery and pharmacy | simple | noTimeFE | -8.423 | 0.007 | -0.015 | 0.001 | -0.016 | -0.013 | 0.000 | 0.13 | 0.02 | 10419 | 10417 |
| Drugs | grocery and pharmacy | afe | timeFE | -7.938 | 0.061 | 0.001 | 0.001 | 0.000 | 0.002 | 0.220 | 0.82 | 0.00 | 10419 | 10061 |
| Drugs | grocery and pharmacy | simple | timeFE | -8.478 | 0.041 | -0.019 | 0.002 | -0.023 | -0.015 | 0.000 | 0.17 | 0.01 | 10419 | 10386 |
| Drugs | parks | afe | noTimeFE | -7.918 | 0.067 | 0.000 | 0.000 | -0.001 | 0.000 | 0.000 | 0.78 | 0.00 | 10329 | 10002 |
| Drugs | parks | simple | noTimeFE | -8.319 | 0.015 | -0.002 | 0.000 | -0.003 | -0.002 | 0.000 | 0.03 | 0.01 | 10329 | 10327 |
| Drugs | parks | afe | timeFE | -7.935 | 0.062 | 0.000 | 0.000 | 0.000 | 0.000 | 0.728 | 0.82 | 0.00 | 10329 | 9971 |
| Drugs | parks | simple | timeFE | -8.428 | 0.045 | -0.003 | 0.000 | -0.003 | -0.002 | 0.000 | 0.06 | 0.01 | 10329 | 10296 |
| Drugs | transit stations | afe | noTimeFE | -8.016 | 0.066 | -0.004 | 0.000 | -0.005 | -0.004 | 0.000 | 0.79 | 0.04 | 10419 | 10092 |
| Drugs | transit stations | simple | noTimeFE | -8.712 | 0.014 | -0.010 | 0.001 | -0.011 | -0.009 | 0.000 | 0.10 | 0.04 | 10419 | 10417 |
| Drugs | transit stations | afe | timeFE | -7.935 | 0.061 | 0.000 | 0.000 | 0.000 | 0.001 | 0.376 | 0.82 | 0.00 | 10419 | 10061 |
| Drugs | transit stations | simple | timeFE | -8.749 | 0.044 | -0.012 | 0.001 | -0.013 | -0.011 | 0.000 | 0.13 | 0.03 | 10419 | 10386 |
| Drugs | workplaces | afe | noTimeFE | -8.083 | 0.068 | -0.008 | 0.000 | -0.008 | -0.007 | 0.000 | 0.80 | 0.07 | 10419 | 10092 |
| Drugs | workplaces | simple | noTimeFE | -8.891 | 0.025 | -0.016 | 0.001 | -0.017 | -0.014 | 0.000 | 0.09 | 0.03 | 10419 | 10417 |
| Drugs | workplaces | afe | timeFE | -7.929 | 0.062 | 0.001 | 0.001 | -0.001 | 0.003 | 0.436 | 0.82 | 0.00 | 10419 | 10061 |
| Drugs | workplaces | simple | timeFE | -9.106 | 0.059 | -0.029 | 0.002 | -0.033 | -0.025 | 0.000 | 0.14 | 0.02 | 10419 | 10386 |
| Drugs | residential | afe | noTimeFE | -8.038 | 0.066 | 0.019 | 0.001 | 0.018 | 0.020 | 0.000 | 0.79 | 0.10 | 10382 | 10055 |
| Drugs | residential | simple | noTimeFE | -8.633 | 0.014 | 0.022 | 0.001 | 0.020 | 0.025 | 0.000 | 0.04 | 0.03 | 10382 | 10380 |
| Drugs | residential | afe | timeFE | -8.005 | 0.063 | 0.010 | 0.003 | 0.005 | 0.015 | 0.000 | 0.81 | 0.00 | 10382 | 10024 |
| Drugs | residential | simple | timeFE | -8.832 | 0.047 | 0.041 | 0.004 | 0.034 | 0.047 | 0.000 | 0.06 | 0.01 | 10382 | 10349 |
| Other Crime | retail and recreation | afe | noTimeFE | -7.948 | 0.041 | 0.001 | 0.000 | 0.000 | 0.001 | 0.000 | 0.71 | 0.00 | 10416 | 10089 |
| Other Crime | retail and recreation | simple | noTimeFE | -8.846 | 0.007 | 0.000 | 0.000 | 0.000 | 0.001 | 0.319 | 0.00 | 0.00 | 10416 | 10414 |
| Other Crime | retail and recreation | afe | timeFE | -7.954 | 0.042 | 0.001 | 0.000 | 0.000 | 0.002 | 0.021 | 0.73 | 0.00 | 10416 | 10058 |
| Other Crime | retail and recreation | simple | timeFE | -8.904 | 0.032 | -0.002 | 0.001 | -0.003 | -0.001 | 0.001 | 0.01 | 0.00 | 10416 | 10383 |
| Other Crime | grocery and pharmacy | afe | noTimeFE | -7.964 | 0.041 | 0.002 | 0.000 | 0.001 | 0.002 | 0.000 | 0.71 | 0.01 | 10416 | 10089 |
| Other Crime | grocery and pharmacy | simple | noTimeFE | -8.850 | 0.005 | 0.001 | 0.000 | 0.000 | 0.002 | 0.041 | 0.00 | 0.00 | 10416 | 10414 |
| Other Crime | grocery and pharmacy | afe | timeFE | -7.980 | 0.040 | 0.001 | 0.001 | 0.000 | 0.002 | 0.195 | 0.73 | 0.00 | 10416 | 10058 |
| Other Crime | grocery and pharmacy | simple | timeFE | -8.865 | 0.030 | -0.001 | 0.001 | -0.002 | 0.000 | 0.130 | 0.01 | 0.00 | 10416 | 10383 |
| Other Crime | parks | afe | noTimeFE | -7.949 | 0.041 | 0.001 | 0.000 | 0.001 | 0.001 | 0.000 | 0.72 | 0.01 | 10326 | 9999 |
| Other Crime | parks | simple | noTimeFE | -8.814 | 0.007 | -0.001 | 0.000 | -0.001 | -0.001 | 0.000 | 0.01 | 0.01 | 10326 | 10324 |
| Other Crime | parks | afe | timeFE | -7.971 | 0.041 | 0.000 | 0.000 | 0.000 | 0.000 | 0.067 | 0.73 | 0.00 | 10326 | 9968 |
| Other Crime | parks | simple | timeFE | -8.863 | 0.030 | -0.002 | 0.000 | -0.002 | -0.002 | 0.000 | 0.03 | 0.01 | 10326 | 10293 |
| Other Crime | transit stations | afe | noTimeFE | -7.936 | 0.041 | 0.001 | 0.000 | 0.001 | 0.002 | 0.000 | 0.71 | 0.00 | 10416 | 10089 |
| Other Crime | transit stations | simple | noTimeFE | -8.843 | 0.009 | 0.000 | 0.000 | 0.000 | 0.001 | 0.292 | 0.00 | 0.00 | 10416 | 10414 |
| Other Crime | transit stations | afe | timeFE | -7.980 | 0.040 | 0.000 | 0.000 | -0.001 | 0.001 | 0.758 | 0.73 | 0.00 | 10416 | 10058 |
| Other Crime | transit stations | simple | timeFE | -8.884 | 0.031 | -0.001 | 0.000 | -0.001 | 0.000 | 0.003 | 0.01 | 0.00 | 10416 | 10383 |
| Other Crime | workplaces | afe | noTimeFE | -7.910 | 0.041 | 0.003 | 0.000 | 0.002 | 0.003 | 0.000 | 0.72 | 0.01 | 10416 | 10089 |
| Other Crime | workplaces | simple | noTimeFE | -8.599 | 0.014 | 0.008 | 0.000 | 0.008 | 0.009 | 0.000 | 0.04 | 0.03 | 10416 | 10414 |
| Other Crime | workplaces | afe | timeFE | -7.989 | 0.042 | -0.001 | 0.001 | -0.002 | 0.002 | 0.636 | 0.73 | 0.00 | 10416 | 10058 |
| Other Crime | workplaces | simple | timeFE | -8.392 | 0.033 | 0.021 | 0.001 | 0.020 | 0.023 | 0.000 | 0.09 | 0.07 | 10416 | 10383 |
| Other Crime | residential | afe | noTimeFE | -7.938 | 0.041 | -0.004 | 0.001 | -0.005 | -0.003 | 0.000 | 0.71 | 0.01 | 10379 | 10052 |
| Other Crime | residential | simple | noTimeFE | -8.705 | 0.010 | -0.016 | 0.001 | -0.018 | -0.015 | 0.000 | 0.03 | 0.03 | 10379 | 10377 |
| Other Crime | residential | afe | timeFE | -7.983 | 0.044 | 0.000 | 0.003 | -0.005 | 0.006 | 0.980 | 0.73 | 0.00 | 10379 | 10021 |
| Other Crime | residential | simple | timeFE | -8.126 | 0.034 | -0.084 | 0.002 | -0.088 | -0.080 | 0.000 | 0.13 | 0.13 | 10379 | 10346 |
| Other Theft | retail and recreation | afe | noTimeFE | -7.006 | 0.029 | 0.006 | 0.000 | 0.006 | 0.006 | 0.000 | 0.87 | 0.28 | 10431 | 10104 |
| Other Theft | retail and recreation | simple | noTimeFE | -7.560 | 0.006 | -0.001 | 0.000 | -0.002 | -0.001 | 0.001 | 0.00 | 0.00 | 10431 | 10429 |
| Other Theft | retail and recreation | afe | timeFE | -7.117 | 0.035 | 0.002 | 0.000 | 0.001 | 0.002 | 0.000 | 0.89 | 0.01 | 10431 | 10073 |
| Other Theft | retail and recreation | simple | timeFE | -7.953 | 0.078 | -0.016 | 0.002 | -0.020 | -0.011 | 0.000 | 0.33 | 0.00 | 10431 | 10398 |
| Other Theft | grocery and pharmacy | afe | noTimeFE | -7.156 | 0.029 | 0.010 | 0.000 | 0.010 | 0.010 | 0.000 | 0.86 | 0.22 | 10431 | 10104 |
| Other Theft | grocery and pharmacy | simple | noTimeFE | -7.538 | 0.008 | -0.007 | 0.001 | -0.010 | -0.004 | 0.000 | 0.05 | 0.00 | 10431 | 10429 |
| Other Theft | grocery and pharmacy | afe | timeFE | -7.170 | 0.035 | 0.002 | 0.000 | 0.001 | 0.003 | 0.000 | 0.89 | 0.00 | 10431 | 10073 |
| Other Theft | grocery and pharmacy | simple | timeFE | -7.580 | 0.050 | -0.018 | 0.002 | -0.022 | -0.013 | 0.000 | 0.33 | 0.01 | 10431 | 10398 |
| Other Theft | parks | afe | noTimeFE | -7.127 | 0.029 | 0.002 | 0.000 | 0.002 | 0.002 | 0.000 | 0.81 | 0.07 | 10341 | 10014 |
| Other Theft | parks | simple | noTimeFE | -7.473 | 0.018 | -0.002 | 0.000 | -0.002 | -0.001 | 0.000 | 0.03 | 0.00 | 10341 | 10339 |
| Other Theft | parks | afe | timeFE | -7.152 | 0.035 | 0.000 | 0.000 | 0.000 | 0.001 | 0.000 | 0.89 | 0.00 | 10341 | 9983 |
| Other Theft | parks | simple | timeFE | -7.519 | 0.068 | -0.003 | 0.000 | -0.004 | -0.002 | 0.000 | 0.18 | 0.01 | 10341 | 10308 |
| Other Theft | transit stations | afe | noTimeFE | -6.977 | 0.029 | 0.008 | 0.000 | 0.008 | 0.008 | 0.000 | 0.86 | 0.24 | 10431 | 10104 |
| Other Theft | transit stations | simple | noTimeFE | -7.553 | 0.012 | -0.001 | 0.000 | -0.001 | 0.000 | 0.034 | 0.00 | 0.00 | 10431 | 10429 |
| Other Theft | transit stations | afe | timeFE | -7.121 | 0.034 | 0.003 | 0.000 | 0.002 | 0.003 | 0.000 | 0.89 | 0.02 | 10431 | 10073 |
| Other Theft | transit stations | simple | timeFE | -7.714 | 0.064 | -0.008 | 0.001 | -0.009 | -0.006 | 0.000 | 0.18 | 0.02 | 10431 | 10398 |
| Other Theft | workplaces | afe | noTimeFE | -6.903 | 0.031 | 0.012 | 0.000 | 0.012 | 0.013 | 0.000 | 0.86 | 0.24 | 10431 | 10104 |
| Other Theft | workplaces | simple | noTimeFE | -7.558 | 0.020 | -0.001 | 0.001 | -0.002 | 0.001 | 0.252 | 0.00 | 0.00 | 10431 | 10429 |
| Other Theft | workplaces | afe | timeFE | -7.083 | 0.036 | 0.007 | 0.001 | 0.005 | 0.008 | 0.000 | 0.89 | 0.01 | 10431 | 10073 |
| Other Theft | workplaces | simple | timeFE | -8.095 | 0.063 | -0.025 | 0.002 | -0.029 | -0.021 | 0.000 | 0.24 | 0.01 | 10431 | 10398 |
| Other Theft | residential | afe | noTimeFE | -6.983 | 0.028 | -0.028 | 0.000 | -0.029 | -0.027 | 0.000 | 0.87 | 0.35 | 10394 | 10067 |
| Other Theft | residential | simple | noTimeFE | -7.389 | 0.013 | -0.020 | 0.001 | -0.022 | -0.017 | 0.000 | 0.05 | 0.03 | 10394 | 10392 |
| Other Theft | residential | afe | timeFE | -6.987 | 0.035 | -0.029 | 0.002 | -0.033 | -0.025 | 0.000 | 0.88 | 0.02 | 10394 | 10036 |
| Other Theft | residential | simple | timeFE | -7.904 | 0.040 | 0.035 | 0.003 | 0.029 | 0.042 | 0.000 | 0.16 | 0.01 | 10394 | 10361 |
| Weapon Possession | retail and recreation | afe | noTimeFE | -9.109 | 0.070 | 0.003 | 0.000 | 0.002 | 0.003 | 0.000 | 0.64 | 0.02 | 10153 | 9826 |
| Weapon Possession | retail and recreation | simple | noTimeFE | -9.725 | 0.009 | 0.000 | 0.000 | -0.001 | 0.001 | 0.954 | 0.00 | 0.00 | 10153 | 10151 |
| Weapon Possession | retail and recreation | afe | timeFE | -9.165 | 0.074 | 0.001 | 0.001 | 0.000 | 0.002 | 0.025 | 0.66 | 0.00 | 10153 | 9795 |
| Weapon Possession | retail and recreation | simple | timeFE | -10.000 | 0.038 | -0.009 | 0.001 | -0.011 | -0.008 | 0.000 | 0.06 | 0.02 | 10153 | 10120 |
| Weapon Possession | grocery and pharmacy | afe | noTimeFE | -9.176 | 0.070 | 0.004 | 0.000 | 0.004 | 0.005 | 0.000 | 0.64 | 0.02 | 10153 | 9826 |
| Weapon Possession | grocery and pharmacy | simple | noTimeFE | -9.725 | 0.007 | 0.000 | 0.000 | -0.001 | 0.001 | 0.759 | 0.00 | 0.00 | 10153 | 10151 |
| Weapon Possession | grocery and pharmacy | afe | timeFE | -9.199 | 0.072 | 0.002 | 0.001 | 0.001 | 0.003 | 0.007 | 0.66 | 0.00 | 10153 | 9795 |
| Weapon Possession | grocery and pharmacy | simple | timeFE | -9.770 | 0.033 | -0.007 | 0.001 | -0.008 | -0.005 | 0.000 | 0.04 | 0.01 | 10153 | 10120 |
| Weapon Possession | parks | afe | noTimeFE | -9.154 | 0.067 | 0.001 | 0.000 | 0.001 | 0.001 | 0.000 | 0.63 | 0.01 | 10063 | 9736 |
| Weapon Possession | parks | simple | noTimeFE | -9.703 | 0.008 | -0.001 | 0.000 | -0.001 | -0.001 | 0.000 | 0.00 | 0.00 | 10063 | 10061 |
| Weapon Possession | parks | afe | timeFE | -9.195 | 0.072 | 0.000 | 0.000 | 0.000 | 0.000 | 0.412 | 0.66 | 0.00 | 10063 | 9705 |
| Weapon Possession | parks | simple | timeFE | -9.755 | 0.033 | -0.002 | 0.000 | -0.002 | -0.002 | 0.000 | 0.04 | 0.01 | 10063 | 10030 |
| Weapon Possession | transit stations | afe | noTimeFE | -9.076 | 0.068 | 0.004 | 0.000 | 0.004 | 0.005 | 0.000 | 0.65 | 0.03 | 10153 | 9826 |
| Weapon Possession | transit stations | simple | noTimeFE | -9.710 | 0.013 | 0.001 | 0.000 | 0.000 | 0.001 | 0.145 | 0.00 | 0.00 | 10153 | 10151 |
| Weapon Possession | transit stations | afe | timeFE | -9.169 | 0.071 | 0.002 | 0.001 | 0.001 | 0.003 | 0.000 | 0.66 | 0.00 | 10153 | 9795 |
| Weapon Possession | transit stations | simple | timeFE | -9.833 | 0.035 | -0.003 | 0.000 | -0.004 | -0.002 | 0.000 | 0.03 | 0.01 | 10153 | 10120 |
| Weapon Possession | workplaces | afe | noTimeFE | -9.071 | 0.070 | 0.005 | 0.000 | 0.004 | 0.006 | 0.000 | 0.64 | 0.02 | 10153 | 9826 |
| Weapon Possession | workplaces | simple | noTimeFE | -9.528 | 0.018 | 0.007 | 0.001 | 0.005 | 0.008 | 0.000 | 0.02 | 0.01 | 10153 | 10151 |
| Weapon Possession | workplaces | afe | timeFE | -9.216 | 0.075 | -0.001 | 0.002 | -0.004 | 0.002 | 0.546 | 0.66 | 0.00 | 10153 | 9795 |
| Weapon Possession | workplaces | simple | timeFE | -9.576 | 0.040 | 0.008 | 0.001 | 0.006 | 0.010 | 0.000 | 0.03 | 0.01 | 10153 | 10120 |
| Weapon Possession | residential | afe | noTimeFE | -9.104 | 0.068 | -0.012 | 0.001 | -0.014 | -0.010 | 0.000 | 0.64 | 0.02 | 10120 | 9793 |
| Weapon Possession | residential | simple | noTimeFE | -9.557 | 0.014 | -0.019 | 0.001 | -0.022 | -0.017 | 0.000 | 0.03 | 0.02 | 10120 | 10118 |
| Weapon Possession | residential | afe | timeFE | -9.320 | 0.082 | 0.017 | 0.004 | 0.009 | 0.025 | 0.000 | 0.66 | 0.00 | 10120 | 9762 |
| Weapon Possession | residential | simple | timeFE | -9.322 | 0.042 | -0.048 | 0.003 | -0.054 | -0.043 | 0.000 | 0.05 | 0.03 | 10120 | 10087 |
| Public Order | retail and recreation | afe | noTimeFE | -6.621 | 0.041 | 0.006 | 0.000 | 0.005 | 0.006 | 0.000 | 0.82 | 0.22 | 10431 | 10104 |
| Public Order | retail and recreation | simple | noTimeFE | -7.249 | 0.006 | 0.002 | 0.000 | 0.002 | 0.003 | 0.000 | 0.01 | 0.01 | 10431 | 10429 |
| Public Order | retail and recreation | afe | timeFE | -6.903 | 0.035 | 0.003 | 0.000 | 0.003 | 0.004 | 0.000 | 0.87 | 0.01 | 10431 | 10073 |
| Public Order | retail and recreation | simple | timeFE | -7.724 | 0.035 | -0.008 | 0.001 | -0.009 | -0.006 | 0.000 | 0.15 | 0.01 | 10431 | 10398 |
| Public Order | grocery and pharmacy | afe | noTimeFE | -6.757 | 0.038 | 0.010 | 0.000 | 0.009 | 0.010 | 0.000 | 0.82 | 0.21 | 10431 | 10104 |
| Public Order | grocery and pharmacy | simple | noTimeFE | -7.290 | 0.005 | 0.002 | 0.001 | 0.001 | 0.003 | 0.001 | 0.00 | 0.00 | 10431 | 10429 |
| Public Order | grocery and pharmacy | afe | timeFE | -6.988 | 0.033 | 0.004 | 0.000 | 0.003 | 0.005 | 0.000 | 0.87 | 0.02 | 10431 | 10073 |
| Public Order | grocery and pharmacy | simple | timeFE | -7.539 | 0.029 | -0.007 | 0.001 | -0.008 | -0.005 | 0.000 | 0.13 | 0.00 | 10431 | 10398 |
| Public Order | parks | afe | noTimeFE | -6.672 | 0.047 | 0.003 | 0.000 | 0.003 | 0.003 | 0.000 | 0.81 | 0.16 | 10341 | 10014 |
| Public Order | parks | simple | noTimeFE | -7.289 | 0.008 | 0.000 | 0.000 | 0.000 | 0.000 | 0.462 | 0.00 | 0.00 | 10341 | 10339 |
| Public Order | parks | afe | timeFE | -6.954 | 0.034 | 0.001 | 0.000 | 0.001 | 0.001 | 0.000 | 0.87 | 0.01 | 10341 | 9983 |
| Public Order | parks | simple | timeFE | -7.524 | 0.029 | -0.002 | 0.000 | -0.002 | -0.002 | 0.000 | 0.13 | 0.01 | 10341 | 10308 |
| Public Order | transit stations | afe | noTimeFE | -6.596 | 0.045 | 0.007 | 0.000 | 0.007 | 0.007 | 0.000 | 0.81 | 0.20 | 10431 | 10104 |
| Public Order | transit stations | simple | noTimeFE | -7.268 | 0.009 | 0.001 | 0.000 | 0.000 | 0.001 | 0.002 | 0.00 | 0.00 | 10431 | 10429 |
| Public Order | transit stations | afe | timeFE | -6.985 | 0.034 | 0.001 | 0.000 | 0.000 | 0.001 | 0.000 | 0.87 | 0.00 | 10431 | 10073 |
| Public Order | transit stations | simple | timeFE | -7.641 | 0.030 | -0.005 | 0.000 | -0.005 | -0.004 | 0.000 | 0.13 | 0.02 | 10431 | 10398 |
| Public Order | workplaces | afe | noTimeFE | -6.573 | 0.042 | 0.009 | 0.000 | 0.009 | 0.009 | 0.000 | 0.79 | 0.14 | 10431 | 10104 |
| Public Order | workplaces | simple | noTimeFE | -7.086 | 0.015 | 0.007 | 0.001 | 0.006 | 0.008 | 0.000 | 0.03 | 0.02 | 10431 | 10429 |
| Public Order | workplaces | afe | timeFE | -7.012 | 0.034 | -0.001 | 0.001 | -0.002 | 0.001 | 0.238 | 0.87 | 0.00 | 10431 | 10073 |
| Public Order | workplaces | simple | timeFE | -7.513 | 0.039 | 0.000 | 0.001 | -0.002 | 0.003 | 0.731 | 0.11 | 0.00 | 10431 | 10398 |
| Public Order | residential | afe | noTimeFE | -6.614 | 0.041 | -0.023 | 0.000 | -0.024 | -0.022 | 0.000 | 0.81 | 0.22 | 10394 | 10067 |
| Public Order | residential | simple | noTimeFE | -7.071 | 0.009 | -0.026 | 0.001 | -0.028 | -0.024 | 0.000 | 0.08 | 0.08 | 10394 | 10392 |
| Public Order | residential | afe | timeFE | -7.043 | 0.034 | 0.006 | 0.002 | 0.003 | 0.010 | 0.000 | 0.87 | 0.00 | 10394 | 10036 |
| Public Order | residential | simple | timeFE | -7.240 | 0.033 | -0.033 | 0.002 | -0.037 | -0.029 | 0.000 | 0.13 | 0.02 | 10394 | 10361 |
| Robbery | retail and recreation | afe | noTimeFE | -9.309 | 0.065 | 0.005 | 0.000 | 0.005 | 0.006 | 0.000 | 0.88 | 0.07 | 9602 | 9275 |
| Robbery | retail and recreation | simple | noTimeFE | -9.753 | 0.014 | -0.010 | 0.001 | -0.011 | -0.009 | 0.000 | 0.05 | 0.04 | 9602 | 9600 |
| Robbery | retail and recreation | afe | timeFE |  |  |  |  |  |  |  |  |  |  |  |
| Robbery | retail and recreation | simple | timeFE |  |  |  |  |  |  |  |  |  |  |  |
| Robbery | grocery and pharmacy | afe | noTimeFE | -9.436 | 0.063 | 0.008 | 0.000 | 0.007 | 0.009 | 0.000 | 0.87 | 0.04 | 9602 | 9275 |
| Robbery | grocery and pharmacy | simple | noTimeFE | -9.575 | 0.012 | -0.019 | 0.001 | -0.021 | -0.017 | 0.000 | 0.08 | 0.04 | 9602 | 9600 |
| Robbery | grocery and pharmacy | afe | timeFE |  |  |  |  |  |  |  |  |  |  |  |
| Robbery | grocery and pharmacy | simple | timeFE |  |  |  |  |  |  |  |  |  |  |  |
| Robbery | parks | afe | noTimeFE | -9.412 | 0.065 | 0.001 | 0.000 | 0.001 | 0.002 | 0.000 | 0.87 | 0.01 | 9519 | 9192 |
| Robbery | parks | simple | noTimeFE | -9.438 | 0.020 | -0.003 | 0.000 | -0.004 | -0.002 | 0.000 | 0.02 | 0.01 | 9519 | 9517 |
| Robbery | parks | afe | timeFE |  |  |  |  |  |  |  |  |  |  |  |
| Robbery | parks | simple | timeFE |  |  |  |  |  |  |  |  |  |  |  |
| Robbery | transit stations | afe | noTimeFE | -9.289 | 0.065 | 0.006 | 0.000 | 0.006 | 0.007 | 0.000 | 0.88 | 0.06 | 9602 | 9275 |
| Robbery | transit stations | simple | noTimeFE | -9.854 | 0.022 | -0.010 | 0.001 | -0.011 | -0.009 | 0.000 | 0.04 | 0.03 | 9602 | 9600 |
| Robbery | transit stations | afe | timeFE |  |  |  |  |  |  |  |  |  |  |  |
| Robbery | transit stations | simple | timeFE |  |  |  |  |  |  |  |  |  |  |  |
| Robbery | workplaces | afe | noTimeFE | -9.226 | 0.065 | 0.010 | 0.000 | 0.009 | 0.011 | 0.000 | 0.88 | 0.06 | 9602 | 9275 |
| Robbery | workplaces | simple | noTimeFE | -10.041 | 0.030 | -0.016 | 0.001 | -0.018 | -0.014 | 0.000 | 0.04 | 0.03 | 9602 | 9600 |
| Robbery | workplaces | afe | timeFE |  |  |  |  |  |  |  |  |  |  |  |
| Robbery | workplaces | simple | timeFE |  |  |  |  |  |  |  |  |  |  |  |
| Robbery | residential | afe | noTimeFE | -9.314 | 0.063 | -0.020 | 0.001 | -0.021 | -0.018 | 0.000 | 0.88 | 0.07 | 9570 | 9243 |
| Robbery | residential | simple | noTimeFE | -9.588 | 0.025 | 0.005 | 0.002 | 0.000 | 0.009 | 0.038 | 0.00 | 0.00 | 9570 | 9568 |
| Robbery | residential | afe | timeFE |  |  |  |  |  |  |  |  |  |  |  |
| Robbery | residential | simple | timeFE |  |  |  |  |  |  |  |  |  |  |  |
| Shoplifting | retail and recreation | afe | noTimeFE | -6.923 | 0.046 | 0.006 | 0.000 | 0.006 | 0.007 | 0.000 | 0.79 | 0.17 | 10423 | 10096 |
| Shoplifting | retail and recreation | simple | noTimeFE | -7.950 | 0.009 | 0.000 | 0.000 | -0.001 | 0.001 | 0.454 | 0.00 | 0.00 | 10423 | 10421 |
| Shoplifting | retail and recreation | afe | timeFE | -7.014 | 0.048 | 0.001 | 0.000 | 0.000 | 0.001 | 0.182 | 0.81 | 0.00 | 10423 | 10065 |
| Shoplifting | retail and recreation | simple | timeFE | -8.300 | 0.067 | -0.015 | 0.002 | -0.019 | -0.011 | 0.000 | 0.18 | 0.01 | 10423 | 10390 |
| Shoplifting | grocery and pharmacy | afe | noTimeFE | -7.077 | 0.043 | 0.011 | 0.000 | 0.010 | 0.011 | 0.000 | 0.78 | 0.15 | 10423 | 10096 |
| Shoplifting | grocery and pharmacy | simple | noTimeFE | -7.944 | 0.007 | -0.004 | 0.001 | -0.006 | -0.001 | 0.002 | 0.01 | 0.00 | 10423 | 10421 |
| Shoplifting | grocery and pharmacy | afe | timeFE | -7.019 | 0.047 | 0.004 | 0.001 | 0.003 | 0.004 | 0.000 | 0.81 | 0.01 | 10423 | 10065 |
| Shoplifting | grocery and pharmacy | simple | timeFE | -7.931 | 0.040 | -0.015 | 0.002 | -0.019 | -0.011 | 0.000 | 0.15 | 0.01 | 10423 | 10390 |
| Shoplifting | parks | afe | noTimeFE | -7.049 | 0.049 | 0.001 | 0.000 | 0.001 | 0.002 | 0.000 | 0.75 | 0.02 | 10333 | 10006 |
| Shoplifting | parks | simple | noTimeFE | -7.879 | 0.014 | -0.002 | 0.000 | -0.003 | -0.002 | 0.000 | 0.02 | 0.01 | 10333 | 10331 |
| Shoplifting | parks | afe | timeFE | -7.010 | 0.047 | 0.000 | 0.000 | 0.000 | 0.001 | 0.001 | 0.81 | 0.00 | 10333 | 9975 |
| Shoplifting | parks | simple | timeFE | -7.891 | 0.045 | -0.003 | 0.000 | -0.004 | -0.003 | 0.000 | 0.12 | 0.01 | 10333 | 10300 |
| Shoplifting | transit stations | afe | noTimeFE | -6.920 | 0.049 | 0.007 | 0.000 | 0.006 | 0.007 | 0.000 | 0.77 | 0.11 | 10423 | 10096 |
| Shoplifting | transit stations | simple | noTimeFE | -7.969 | 0.012 | -0.001 | 0.000 | -0.002 | 0.000 | 0.034 | 0.00 | 0.00 | 10423 | 10421 |
| Shoplifting | transit stations | afe | timeFE | -7.042 | 0.047 | -0.001 | 0.000 | -0.001 | 0.000 | 0.018 | 0.81 | 0.00 | 10423 | 10065 |
| Shoplifting | transit stations | simple | timeFE | -8.086 | 0.046 | -0.008 | 0.001 | -0.009 | -0.007 | 0.000 | 0.11 | 0.02 | 10423 | 10390 |
| Shoplifting | workplaces | afe | noTimeFE | -6.780 | 0.044 | 0.014 | 0.000 | 0.014 | 0.015 | 0.000 | 0.79 | 0.20 | 10423 | 10096 |
| Shoplifting | workplaces | simple | noTimeFE | -7.730 | 0.031 | 0.007 | 0.001 | 0.005 | 0.009 | 0.000 | 0.02 | 0.00 | 10423 | 10421 |
| Shoplifting | workplaces | afe | timeFE | -6.998 | 0.049 | 0.002 | 0.001 | 0.000 | 0.004 | 0.027 | 0.81 | 0.00 | 10423 | 10065 |
| Shoplifting | workplaces | simple | timeFE | -8.067 | 0.071 | -0.008 | 0.003 | -0.013 | -0.003 | 0.002 | 0.08 | 0.00 | 10423 | 10390 |
| Shoplifting | residential | afe | noTimeFE | -6.894 | 0.046 | -0.029 | 0.001 | -0.030 | -0.028 | 0.000 | 0.78 | 0.21 | 10387 | 10060 |
| Shoplifting | residential | simple | noTimeFE | -7.695 | 0.012 | -0.031 | 0.001 | -0.033 | -0.029 | 0.000 | 0.08 | 0.08 | 10387 | 10385 |
| Shoplifting | residential | afe | timeFE | -7.082 | 0.051 | 0.008 | 0.003 | 0.002 | 0.013 | 0.007 | 0.79 | 0.00 | 10387 | 10029 |
| Shoplifting | residential | simple | timeFE | -7.679 | 0.041 | -0.027 | 0.003 | -0.033 | -0.022 | 0.000 | 0.09 | 0.01 | 10387 | 10354 |
| Theft Person | retail and recreation | afe | noTimeFE | -9.622 | 0.111 | 0.013 | 0.000 | 0.013 | 0.014 | 0.000 | 0.93 | 0.20 | 9467 | 9140 |
| Theft Person | retail and recreation | simple | noTimeFE | -9.764 | 0.022 | -0.024 | 0.001 | -0.026 | -0.021 | 0.000 | 0.15 | 0.03 | 9467 | 9465 |
| Theft Person | retail and recreation | afe | timeFE |  |  |  |  |  |  |  |  |  |  |  |
| Theft Person | retail and recreation | simple | timeFE |  |  |  |  |  |  |  |  |  |  |  |
| Theft Person | grocery and pharmacy | afe | noTimeFE | -9.933 | 0.107 | 0.020 | 0.001 | 0.019 | 0.021 | 0.000 | 0.92 | 0.16 | 9467 | 9140 |
| Theft Person | grocery and pharmacy | simple | noTimeFE | -9.382 | 0.027 | -0.037 | 0.003 | -0.042 | -0.032 | 0.000 | 0.25 | 0.02 | 9467 | 9465 |
| Theft Person | grocery and pharmacy | afe | timeFE |  |  |  |  |  |  |  |  |  |  |  |
| Theft Person | grocery and pharmacy | simple | timeFE |  |  |  |  |  |  |  |  |  |  |  |
| Theft Person | parks | afe | noTimeFE | -9.902 | 0.110 | 0.002 | 0.000 | 0.001 | 0.002 | 0.000 | 0.89 | 0.01 | 9391 | 9064 |
| Theft Person | parks | simple | noTimeFE | -8.887 | 0.074 | -0.010 | 0.002 | -0.014 | -0.006 | 0.000 | 0.09 | 0.00 | 9391 | 9389 |
| Theft Person | parks | afe | timeFE |  |  |  |  |  |  |  |  |  |  |  |
| Theft Person | parks | simple | timeFE |  |  |  |  |  |  |  |  |  |  |  |
| Theft Person | transit stations | afe | noTimeFE | -9.583 | 0.108 | 0.015 | 0.000 | 0.015 | 0.016 | 0.000 | 0.92 | 0.17 | 9467 | 9140 |
| Theft Person | transit stations | simple | noTimeFE | -9.700 | 0.064 | -0.017 | 0.001 | -0.020 | -0.014 | 0.000 | 0.05 | 0.01 | 9467 | 9465 |
| Theft Person | transit stations | afe | timeFE |  |  |  |  |  |  |  |  |  |  |  |
| Theft Person | transit stations | simple | timeFE |  |  |  |  |  |  |  |  |  |  |  |
| Theft Person | workplaces | afe | noTimeFE | -9.439 | 0.112 | 0.024 | 0.001 | 0.023 | 0.025 | 0.000 | 0.92 | 0.18 | 9467 | 9140 |
| Theft Person | workplaces | simple | noTimeFE | -10.561 | 0.054 | -0.043 | 0.002 | -0.047 | -0.039 | 0.000 | 0.12 | 0.04 | 9467 | 9465 |
| Theft Person | workplaces | afe | timeFE |  |  |  |  |  |  |  |  |  |  |  |
| Theft Person | workplaces | simple | timeFE |  |  |  |  |  |  |  |  |  |  |  |
| Theft Person | residential | afe | noTimeFE | -9.627 | 0.108 | -0.051 | 0.001 | -0.053 | -0.049 | 0.000 | 0.92 | 0.22 | 9436 | 9109 |
| Theft Person | residential | simple | noTimeFE | -9.362 | 0.064 | 0.003 | 0.005 | -0.007 | 0.013 | 0.576 | 0.00 | 0.00 | 9436 | 9434 |
| Theft Person | residential | afe | timeFE |  |  |  |  |  |  |  |  |  |  |  |
| Theft Person | residential | simple | timeFE |  |  |  |  |  |  |  |  |  |  |  |
| Vehicle Crime | retail and recreation | afe | noTimeFE | -7.649 | 0.050 | 0.003 | 0.000 | 0.003 | 0.004 | 0.000 | 0.83 | 0.06 | 10427 | 10100 |
| Vehicle Crime | retail and recreation | simple | noTimeFE | -7.858 | 0.008 | -0.004 | 0.000 | -0.004 | -0.003 | 0.000 | 0.02 | 0.02 | 10427 | 10425 |
| Vehicle Crime | retail and recreation | afe | timeFE | -7.492 | 0.048 | 0.003 | 0.000 | 0.002 | 0.003 | 0.000 | 0.85 | 0.00 | 10427 | 10069 |
| Vehicle Crime | retail and recreation | simple | timeFE | -8.162 | 0.064 | -0.020 | 0.002 | -0.024 | -0.016 | 0.000 | 0.19 | 0.01 | 10427 | 10394 |
| Vehicle Crime | grocery and pharmacy | afe | noTimeFE | -7.733 | 0.049 | 0.005 | 0.000 | 0.005 | 0.006 | 0.000 | 0.83 | 0.05 | 10427 | 10100 |
| Vehicle Crime | grocery and pharmacy | simple | noTimeFE | -7.784 | 0.007 | -0.009 | 0.001 | -0.010 | -0.008 | 0.000 | 0.04 | 0.03 | 10427 | 10425 |
| Vehicle Crime | grocery and pharmacy | afe | timeFE | -7.565 | 0.046 | 0.003 | 0.001 | 0.002 | 0.004 | 0.000 | 0.85 | 0.00 | 10427 | 10069 |
| Vehicle Crime | grocery and pharmacy | simple | timeFE | -7.661 | 0.035 | -0.020 | 0.002 | -0.023 | -0.017 | 0.000 | 0.15 | 0.02 | 10427 | 10394 |
| Vehicle Crime | parks | afe | noTimeFE | -7.734 | 0.051 | 0.000 | 0.000 | 0.000 | 0.000 | 0.015 | 0.82 | 0.00 | 10337 | 10010 |
| Vehicle Crime | parks | simple | noTimeFE | -7.694 | 0.009 | -0.003 | 0.000 | -0.003 | -0.002 | 0.000 | 0.03 | 0.02 | 10337 | 10335 |
| Vehicle Crime | parks | afe | timeFE | -7.543 | 0.047 | 0.001 | 0.000 | 0.000 | 0.001 | 0.000 | 0.85 | 0.00 | 10337 | 9979 |
| Vehicle Crime | parks | simple | timeFE | -7.622 | 0.035 | -0.003 | 0.000 | -0.003 | -0.003 | 0.000 | 0.06 | 0.02 | 10337 | 10304 |
| Vehicle Crime | transit stations | afe | noTimeFE | -7.642 | 0.052 | 0.004 | 0.000 | 0.004 | 0.004 | 0.000 | 0.82 | 0.05 | 10427 | 10100 |
| Vehicle Crime | transit stations | simple | noTimeFE | -7.973 | 0.011 | -0.006 | 0.000 | -0.007 | -0.006 | 0.000 | 0.04 | 0.05 | 10427 | 10425 |
| Vehicle Crime | transit stations | afe | timeFE | -7.554 | 0.047 | 0.001 | 0.000 | 0.000 | 0.001 | 0.001 | 0.85 | 0.00 | 10427 | 10069 |
| Vehicle Crime | transit stations | simple | timeFE | -7.975 | 0.034 | -0.013 | 0.000 | -0.014 | -0.012 | 0.000 | 0.14 | 0.08 | 10427 | 10394 |
| Vehicle Crime | workplaces | afe | noTimeFE | -7.563 | 0.050 | 0.008 | 0.000 | 0.008 | 0.009 | 0.000 | 0.83 | 0.08 | 10427 | 10100 |
| Vehicle Crime | workplaces | simple | noTimeFE | -8.004 | 0.017 | -0.008 | 0.001 | -0.009 | -0.006 | 0.000 | 0.02 | 0.02 | 10427 | 10425 |
| Vehicle Crime | workplaces | afe | timeFE | -7.536 | 0.049 | 0.003 | 0.001 | 0.001 | 0.005 | 0.011 | 0.85 | 0.00 | 10427 | 10069 |
| Vehicle Crime | workplaces | simple | timeFE | -8.458 | 0.039 | -0.036 | 0.001 | -0.038 | -0.034 | 0.000 | 0.18 | 0.10 | 10427 | 10394 |
| Vehicle Crime | residential | afe | noTimeFE | -7.642 | 0.048 | -0.015 | 0.001 | -0.016 | -0.014 | 0.000 | 0.83 | 0.08 | 10390 | 10063 |
| Vehicle Crime | residential | simple | noTimeFE | -7.810 | 0.013 | 0.003 | 0.001 | 0.001 | 0.006 | 0.004 | 0.00 | 0.00 | 10390 | 10388 |
| Vehicle Crime | residential | afe | timeFE | -7.561 | 0.050 | -0.002 | 0.003 | -0.007 | 0.003 | 0.472 | 0.85 | 0.00 | 10390 | 10032 |
| Vehicle Crime | residential | simple | timeFE | -8.611 | 0.044 | 0.105 | 0.003 | 0.099 | 0.112 | 0.000 | 0.15 | 0.08 | 10390 | 10357 |
| Violence & Sex Offences | retail and recreation | afe | noTimeFE | -5.289 | 0.020 | 0.004 | 0.000 | 0.004 | 0.004 | 0.000 | 0.90 | 0.36 | 10432 | 10105 |
| Violence & Sex Offences | retail and recreation | simple | noTimeFE | -5.842 | 0.005 | 0.002 | 0.000 | 0.002 | 0.003 | 0.000 | 0.02 | 0.02 | 10432 | 10430 |
| Violence & Sex Offences | retail and recreation | afe | timeFE | -5.449 | 0.020 | 0.002 | 0.000 | 0.002 | 0.003 | 0.000 | 0.92 | 0.02 | 10432 | 10074 |
| Violence & Sex Offences | retail and recreation | simple | timeFE | -6.097 | 0.027 | -0.003 | 0.001 | -0.004 | -0.002 | 0.000 | 0.10 | 0.00 | 10432 | 10399 |
| Violence & Sex Offences | grocery and pharmacy | afe | noTimeFE | -5.390 | 0.018 | 0.007 | 0.000 | 0.007 | 0.007 | 0.000 | 0.89 | 0.31 | 10432 | 10105 |
| Violence & Sex Offences | grocery and pharmacy | simple | noTimeFE | -5.891 | 0.004 | 0.003 | 0.000 | 0.002 | 0.004 | 0.000 | 0.01 | 0.00 | 10432 | 10430 |
| Violence & Sex Offences | grocery and pharmacy | afe | timeFE | -5.511 | 0.018 | 0.003 | 0.000 | 0.003 | 0.003 | 0.000 | 0.92 | 0.02 | 10432 | 10074 |
| Violence & Sex Offences | grocery and pharmacy | simple | timeFE | -6.019 | 0.023 | -0.003 | 0.001 | -0.004 | -0.001 | 0.001 | 0.09 | 0.00 | 10432 | 10399 |
| Violence & Sex Offences | parks | afe | noTimeFE | -5.349 | 0.026 | 0.002 | 0.000 | 0.002 | 0.002 | 0.000 | 0.86 | 0.14 | 10342 | 10015 |
| Violence & Sex Offences | parks | simple | noTimeFE | -5.889 | 0.006 | 0.000 | 0.000 | 0.000 | 0.000 | 0.077 | 0.00 | 0.00 | 10342 | 10340 |
| Violence & Sex Offences | parks | afe | timeFE | -5.483 | 0.019 | 0.001 | 0.000 | 0.001 | 0.001 | 0.000 | 0.92 | 0.02 | 10342 | 9984 |
| Violence & Sex Offences | parks | simple | timeFE | -6.013 | 0.023 | -0.001 | 0.000 | -0.001 | -0.001 | 0.000 | 0.10 | 0.01 | 10342 | 10309 |
| Violence & Sex Offences | transit stations | afe | noTimeFE | -5.283 | 0.024 | 0.005 | 0.000 | 0.005 | 0.005 | 0.000 | 0.88 | 0.23 | 10432 | 10105 |
| Violence & Sex Offences | transit stations | simple | noTimeFE | -5.840 | 0.006 | 0.002 | 0.000 | 0.001 | 0.002 | 0.000 | 0.01 | 0.01 | 10432 | 10430 |
| Violence & Sex Offences | transit stations | afe | timeFE | -5.509 | 0.019 | 0.001 | 0.000 | 0.000 | 0.001 | 0.000 | 0.92 | 0.00 | 10432 | 10074 |
| Violence & Sex Offences | transit stations | simple | timeFE | -6.051 | 0.023 | -0.002 | 0.000 | -0.002 | -0.001 | 0.000 | 0.09 | 0.00 | 10432 | 10399 |
| Violence & Sex Offences | workplaces | afe | noTimeFE | -5.231 | 0.021 | 0.008 | 0.000 | 0.008 | 0.008 | 0.000 | 0.88 | 0.29 | 10432 | 10105 |
| Violence & Sex Offences | workplaces | simple | noTimeFE | -5.621 | 0.010 | 0.009 | 0.000 | 0.008 | 0.010 | 0.000 | 0.08 | 0.06 | 10432 | 10430 |
| Violence & Sex Offences | workplaces | afe | timeFE | -5.503 | 0.020 | 0.001 | 0.000 | 0.000 | 0.002 | 0.008 | 0.92 | 0.00 | 10432 | 10074 |
| Violence & Sex Offences | workplaces | simple | timeFE | -5.791 | 0.029 | 0.010 | 0.001 | 0.008 | 0.012 | 0.000 | 0.12 | 0.01 | 10432 | 10399 |
| Violence & Sex Offences | residential | afe | noTimeFE | -5.283 | 0.020 | -0.017 | 0.000 | -0.018 | -0.017 | 0.000 | 0.89 | 0.36 | 10395 | 10068 |
| Violence & Sex Offences | residential | simple | noTimeFE | -5.681 | 0.007 | -0.024 | 0.001 | -0.026 | -0.023 | 0.000 | 0.13 | 0.14 | 10395 | 10393 |
| Violence & Sex Offences | residential | afe | timeFE | -5.539 | 0.019 | 0.003 | 0.001 | 0.001 | 0.005 | 0.005 | 0.92 | 0.00 | 10395 | 10037 |
| Violence & Sex Offences | residential | simple | timeFE | -5.520 | 0.024 | -0.057 | 0.002 | -0.060 | -0.053 | 0.000 | 0.19 | 0.10 | 10395 | 10362 |
